# Supplementary material for: Interspecific interactions among functionally diverse frugivores and their outcomes for plant reproduction: A new approach based on camera-trap data and tailored null models
Source: PLoS One. 2020 Oct 16;15(10):e0240614. doi: 10.1371/journal.pone.0240614 (PMC7567357; doi:10.1371/journal.pone.0240614)
Supplement: S1 File — A) Red deer plucking the fruits from Pyrus bourgaeana. B) Frugivores not interacting with the plant (value = 0). C) Frugivores physically interacting with the plant, most likely eating its fruits (interaction value = 1). (PDF) [file pone.0240614.s001.pdf]

A)

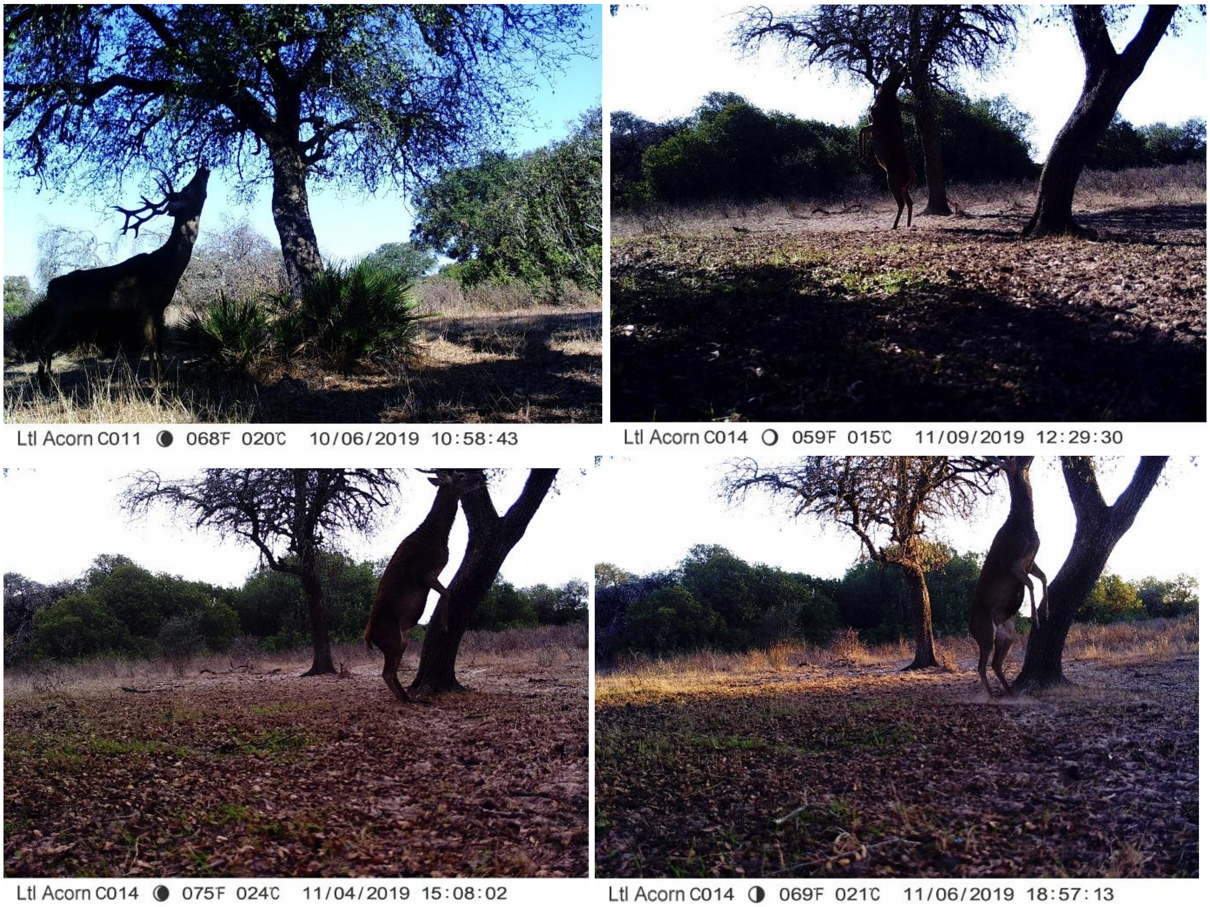
Ltl Acorn C011 068F 020C 10/06/2019 10:58:43
Ltl Acorn C014 059F 015C 11/09/2019 12:29:30
Ltl Acorn C014 075F 024C 11/04/2019 15:08:02
Ltl Acorn C014 069F 021C 11/06/2019 18:57:13

B)

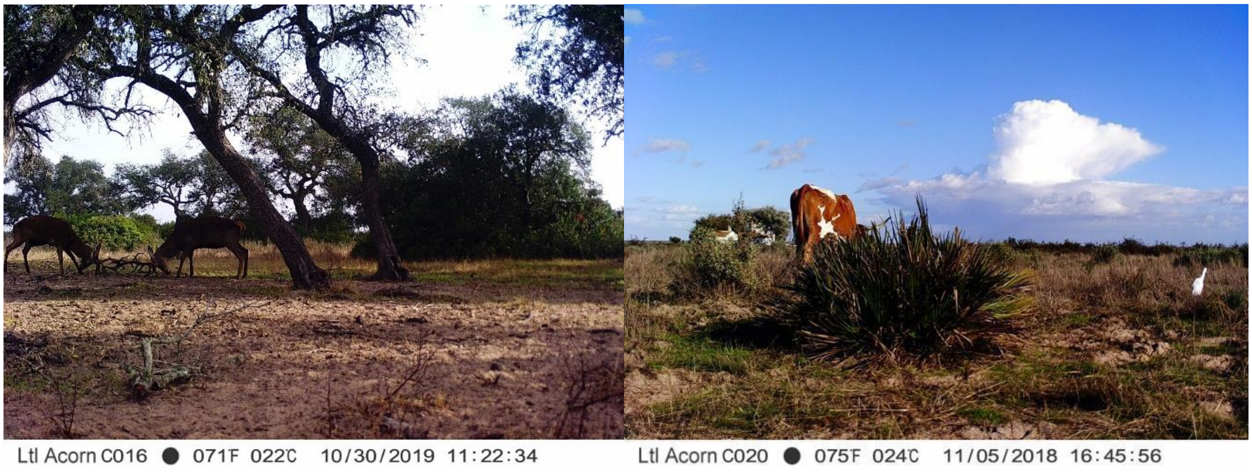
Ltl Acorn C016 071F 022C 10/30/2019 11:22:34
Ltl Acorn C020 075F 024C 11/05/2018 16:45:56

C)

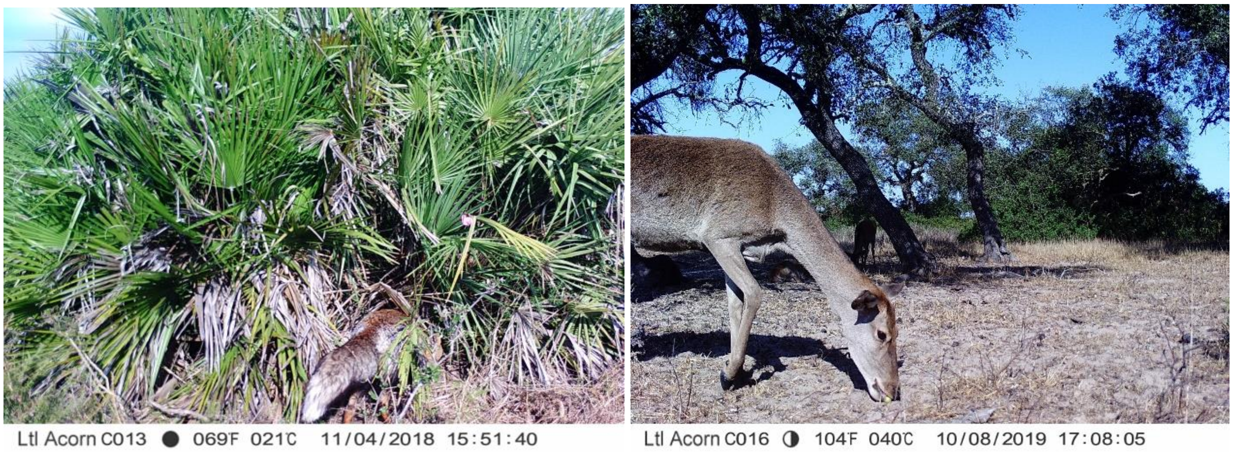
Ltl Acorn C013 069F 021C 11/04/2018 15:51:40
Ltl Acorn C016 104F 040C 10/08/2019 17:08:05
