# Supplementary material for: Interspecific interactions among functionally diverse frugivores and their outcomes for plant reproduction: A new approach based on camera-trap data and tailored null models
Source: PLoS One. 2020 Oct 16;15(10):e0240614. doi: 10.1371/journal.pone.0240614 (PMC7567357; doi:10.1371/journal.pone.0240614)
Supplement: S2 File — Main functions for tailored models 1, 2 and 3 used in the analysis. (PDF) [file pone.0240614.s002.pdf]

**NULL MODEL 1:** This null model was tailored to compare the average number of visits by frugivores of one species (sp1) to the target individual plant visited (PV) and not visited (PNV) by a second frugivore species (sp2).

R Package: dplyr. Mailund, T. (2019). Manipulating Data Frames: dplyr. In *R Data Science Quick Reference* (pp. 109-160). Apress, Berkeley, CA.

```
mat <- matrix(NA, nrow = nrow(sumObs), ncol = nrow(sumObs), dimnames =
list(sumObs$spV1, sumObs$spV1)) #From the observed data, fixes a matrix with the
species to compare.

store<- c() #Generates the storage list where the expected values will be saved.
for (c in 1:ncol(mat)){ #Starts the first loop.
  for (r in 1:nrow(mat)){ #Starts the second loop.
    name_row = rownames(mat)[r]
    name_col = rownames(mat)[c] #Sets the pairs of species from the input matrix.

    ss_row = camCount %>% filter (spV1 == name_row)
    ss_col = camCount %>% filter (spV1 == name_col) #Filters from the input frequency
data each pair of species.

    trap_row = unique(ss_row$camara)
    trap_col = unique(ss_col$camara) #Fixes a unique value for each camera (i.e.
camera trapped palm tree) and its pairs of species frequency.

    intersect_trap = intersect(trap_col, trap_row) #Function to remove cameras which
are shared by both species.

    ss_analysis_row = camCount %>% filter (spV1 %in% c(name_row)) %>% filter (!camara
%in% intersect_trap)
    ss_analysis_col = camCount %>% filter (spV1 %in% c(name_col)) %>% filter (!camara
%in% intersect_trap) #Removes for each pair of species the shared cameras.
    n_total_row = length(unique(ss_analysis_row$camara))
```

```
    n_total_col = length(unique(ss_analysis_col$camara)) #Counts for each non shared camera the frequency per pair of species


    freq_total_row = sum (ss_analysis_row$Freq) / n_total_row

    freq_total_col = sum (ss_analysis_col$Freq) / n_total_col #Sum of pair of species expected frequency and division between total number of nonshared cameras to calculate the average expected frequency of species per camera.


    store<- rbind(store, c(name_row, freq_total_row, name_col))

    store<- rbind(store, c(name_col, freq_total_col, name_row)) #Saves in the "store" that was created earlier the final result of each pair of species average expected frequency per camera with its compared partner.

  }

}

Store #Result in list format.
```

**NULL MODEL 2:** This tailored null model allowed us to calculate the expected minimum time elapsed between pairs of frugivore species which occurred at spatially independent plant individuals.

R package: lubridate. Grolemund, G., & Wickham, H. (2011). Dates and times made easy with lubridate. *Journal of Statistical Software*, 40, 1-25.

```
CalculateMatrix <- function(cam1, cam2) { #Creating the function to calculate expected time differences between independent plants.

Mdiff=matrix(NA, nrow=nrow(cam1), ncol=nrow(cam2),dimnames = list(cam1$spV1, cam2$spV1)) #Generates the storage matrix where time differences will be saved for each pair of species.

  for (i in 1:nrow(cam1) ) { #Starts the first loop. Selects species to compare from camera 1.

    for ( j in 1:nrow(cam2))  { #Starts the second loop. Selects species to compare from camera 2.

      datetime1 <- paste(cam1[i,"date"], cam1[i,"time"])
```

```
      datetime2 <- paste(cam2[j,"date"], cam2[j,"time"]) #Paste our datetime data for each camera.

      Datetime1 <- as.POSIXct(datetime1, format = "%d-%m-%Y %H_%M")

      Datetime2 <- as.POSIXct(datetime2, format = "%d-%m-%Y %H_%M") #Formats our data to work with POSIXct datetime format.

      Mdiff[i,j] <- (as.numeric(Datetime1)-as.numeric(Datetime2))/60 #Calculates the expected time difference between pairs of species (result in minutes). Divide by 60 to obtain result in hours.

            }

        }

     return(Mdiff) #Result in matrix format.

}


firstSp <- function(Mdiff) { #Creates function which will return the time difference of the first visit of a different species (i.e. minimum time elapsed between visits).

  species=unique(colnames(Mdiff)) #Uses the matrix generated above to treat each species as unique.

  mat=matrix(nrow=nrow(Mdiff), ncol=length(species), dimnames = list(row.names(Mdiff),  species)) #Generates the storage matrix where time differences will be saved for the first visit of a different species for each pair.

  for(i in 1:nrow(Mdiff)){ #Starts the first loop.

    for(j in 1:length(species)){ #Starts the second loop. Sets our input data matrix to analyze.

      ss <- as.matrix(Mdiff[,which(colnames(Mdiff)==species[j])]) #Searches for each species individual its position in the matrix.

      if (sum(is.na(ss[i,]))==ncol(ss)){ #Checks if it appears more than once.

        mat[i,j] <- NA #If not the first species, will return NA.

      } else {

        value <- min(ss[i,], na.rm = T) #Returns the time difference of the first visit.

        mat[i,j] <- value #Paste the result in the “storage” matrix.

      }

    }

  }

  return(mat) #Returns result in matrix format.

}
```

**NULL MODEL 3:** This tailored null model allowed us to calculate expected time differences between pairs of frugivore species, based on the randomization of their occurrence at each plant individual.

R package: lubridate. Grolemund, G., & Wickham, H. (2011). Dates and times made easy with lubridate. *Journal of Statistical Software*, 40, 1-25.

R package: plyr. Anderson, S. (2012). A quick introduction to plyr.

```
N <- 1000 #Sets the number of permutations (samples).

Data$daytime1 <- as.POSIXct(paste(Data$day1, Data$time1), format = "%d-%m-%y %H_%M")

Data$daytime2 <- as.POSIXct(paste(Data$day2, Data$time2), format = "%d-%m-%y %H_%M")
#Pastes our datetime data for each camera and formats our data to work with POSIXct
datetime format.


diff <- matrix(NA, nrow(Data)*N, ncol = 5) #Generates the storage matrix where our
sampled data will be saved.

coloriginal <- Data$datetime2 #Fixes the original datetime column to sample.

camoriginal <- Data$cam2 #Fixes the original camera column to sample.


Data$cam1[is.na(Data$cam1)] <- 0 #Paste a 0 value to the camera with NA values as it
has less observations.


k <- 1 #Necessary so each loop result gets saved in a different row.

for (i in 1:N) { #Starts the first loop.

  Data$datetime2 <- sample(coloriginal) #samples the original datetime column.

  Data$cam2 <- sample(camoriginal) #samples the original camera column.

  for(j in 1:nrow(Data)){ #Starts the second loop.

    if ((Data$cam1[j])==(Data$cam2[j])){ #Searches if the sampled datetimes match the
same two cameras where each species occurs.

      diff[k,] <- c(Data$cam1[j], Data$cam2[j], #Matching datetimes at same camera.

                    as.character(Data$daytime1[j]), as.character(Data$daytime2[j]),

                    (as.numeric(Data$daytime1[j])-as.numeric(Data$daytime2[j]))/60)
#Calculates the expected time difference between the pairs of species that randomly
occur at same camera (result in minutes). Divide by 60 to obtain result in hours.
```

```
      k<-k+1 #Saves results.
    }
  }
return(diff) #Returns result in matrix format.
}
file_list <- list.files("C:/Work-directory ") #read files for each pair of species

dataset <- data.frame() #initiate a blank data frame, each iteration of the loop will append the data from the given file to this variable
name <- NULL

#specify columns
for (i in file_list){

  temp_data <- read.csv(i) #each file will be read in, Specify which columns you need read in to avoid any errors
  for (i in i) {
    nam<- rep(i,nrow(temp_data))
    a<- matrix(nam, nrow(temp_data):1)
  }
  name<- rbind(name,a)
  dataset <- rbind(dataset, temp_data) #for each iteration, bind the new data to the building dataset
}

dataset$name <- name #creates one single dataset for the pairs of species
```
