# Supplementary material for: Interspecific interactions among functionally diverse frugivores and their outcomes for plant reproduction: A new approach based on camera-trap data and tailored null models
Source: PLoS One. 2020 Oct 16;15(10):e0240614. doi: 10.1371/journal.pone.0240614 (PMC7567357; doi:10.1371/journal.pone.0240614)

A) **Difference in *Chamaerops humilis* visit patterns for frugivore pairs of species within a time window higher than 5 minutes (null model 1).** The observed values (black bars) represent the mean number of visits by a frugivore species (sp1) to individual plants visited (PV) by a second frugivore species (sp2). The expected values (grey bars) represent the mean number of visits by sp1 to plants not visited (PNV) by sp2. (* P<0.05, ** P<0.01, *** P<0.001).

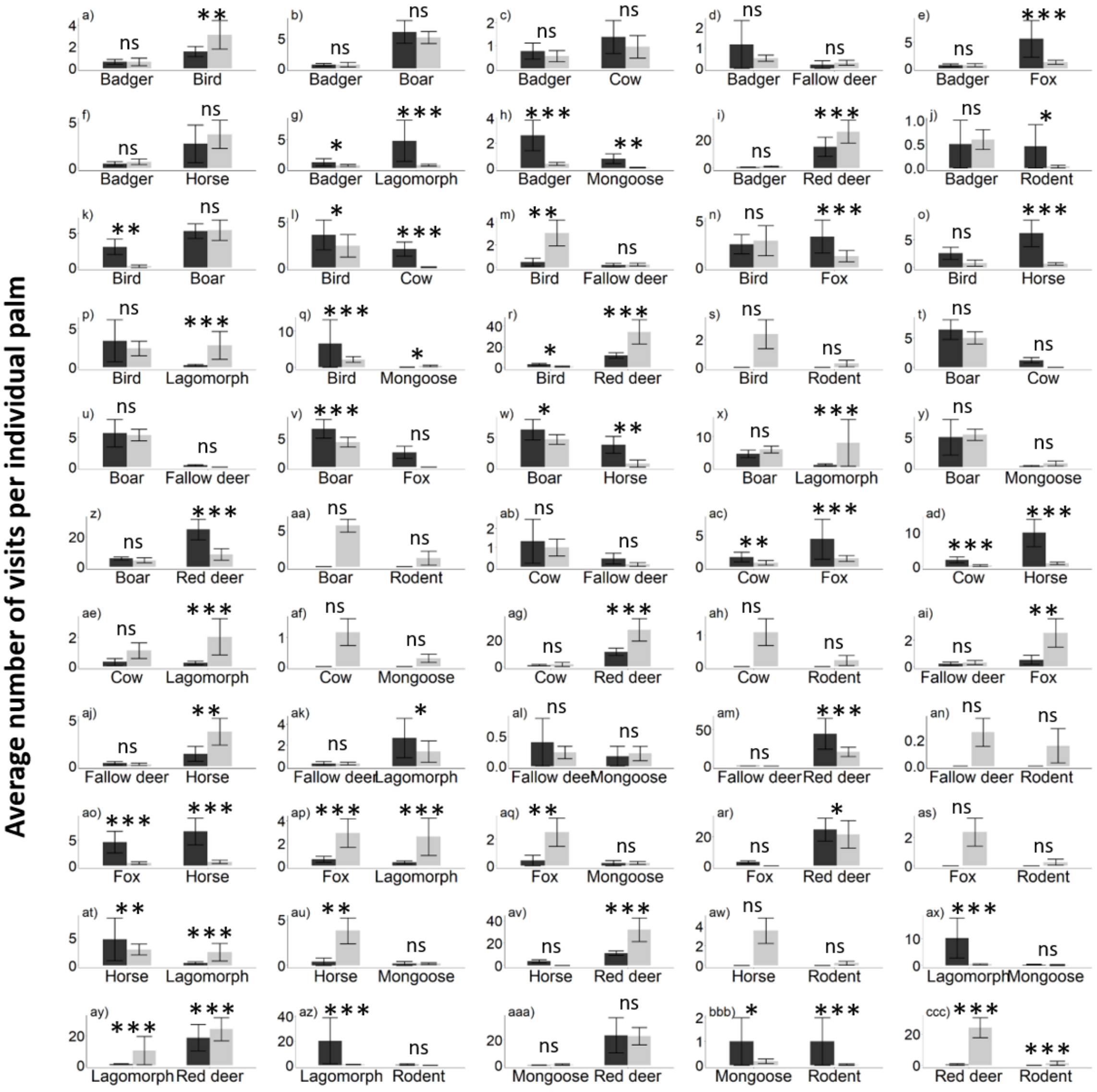

B) **Difference in *Chamaerops humilis* visit patterns for frugivore pairs of species within a time window higher than 30 minutes (null model 1).** The observed values (black bars) represent the mean number of visits by a frugivore species (sp1) to individual plants visited (PV) by a second frugivore species (sp2). The expected values (grey bars) represent the mean number of visits by sp1 to plants not visited (PNV) by sp2. (* *P*<0.05, ** *P*<0.01, *** *P*<0.001).

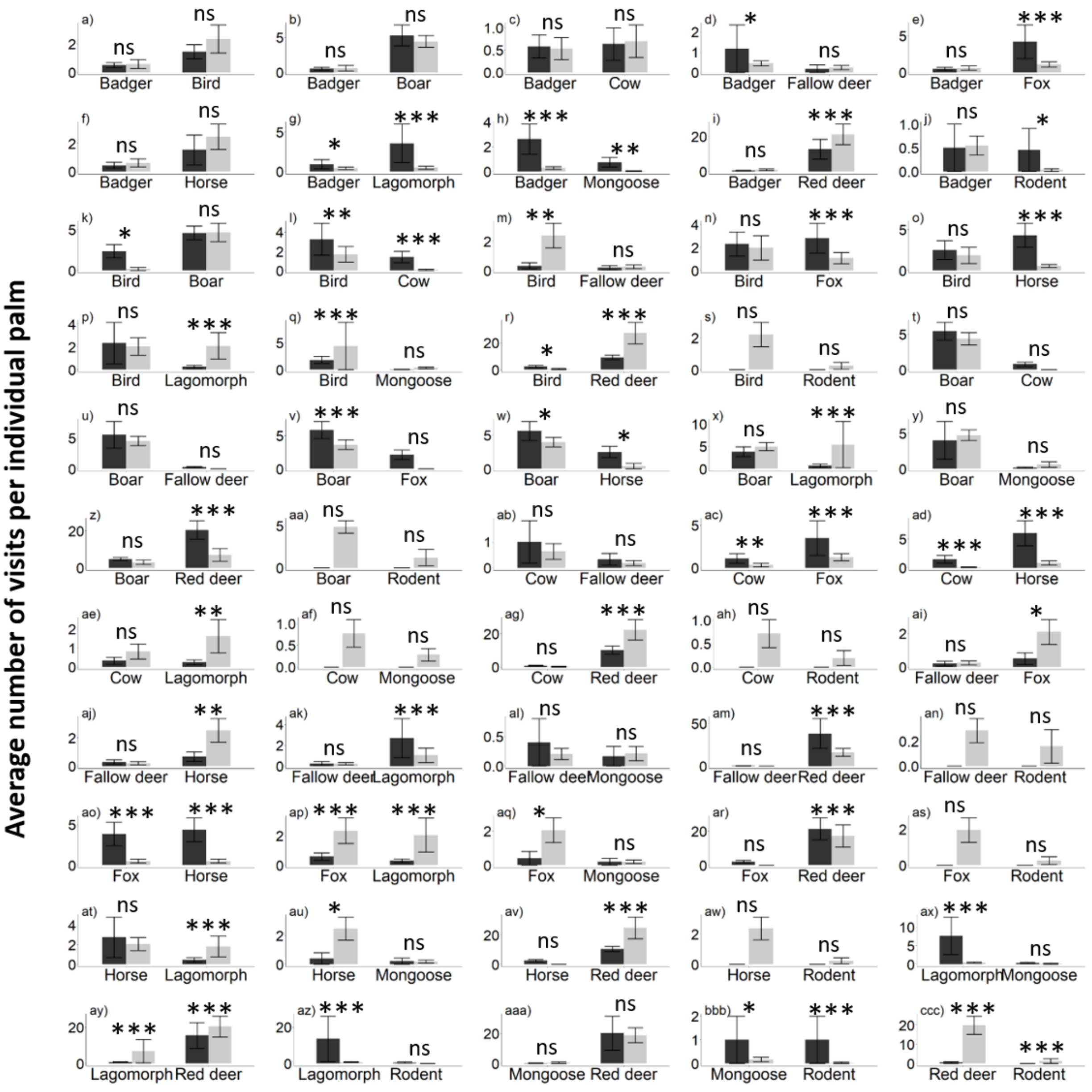

C) **Zoom of difference in *Chamaerops humilis* visit patterns for frugivore pairs of species within a time window higher than 5 minutes (null model 1).** The observed values (black bars) represent the mean number of visits by a frugivore species (sp1) to individual plants visited (PV) by a second frugivore species (sp2). The expected values (grey bars) represent the mean number of visits by sp1 to plants not visited (PNV) by sp2. (* $P<0.05$, ** $P<0.01$, *** $P<0.001$).

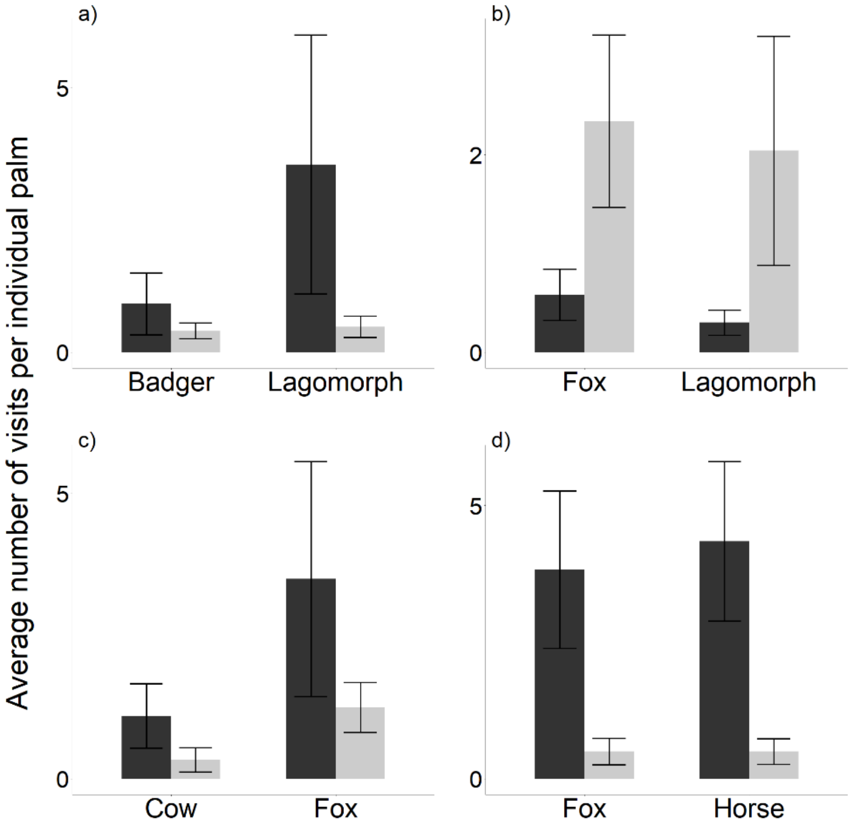

D) **Difference in *P. bourgaeana* visit patterns for frugivore pairs of species within a time window higher than 5 minutes (null model 1).** The observed values (black bars) represent the mean number of visits by a frugivore species (sp1) to individual plants visited (PV) by a second frugivore species (sp2). The expected values (grey bars) represent the mean number of visits by sp1 to plants not visited (PNV) by sp2. (* *P*<0.05, ** *P*<0.01, *** *P*<0.001).

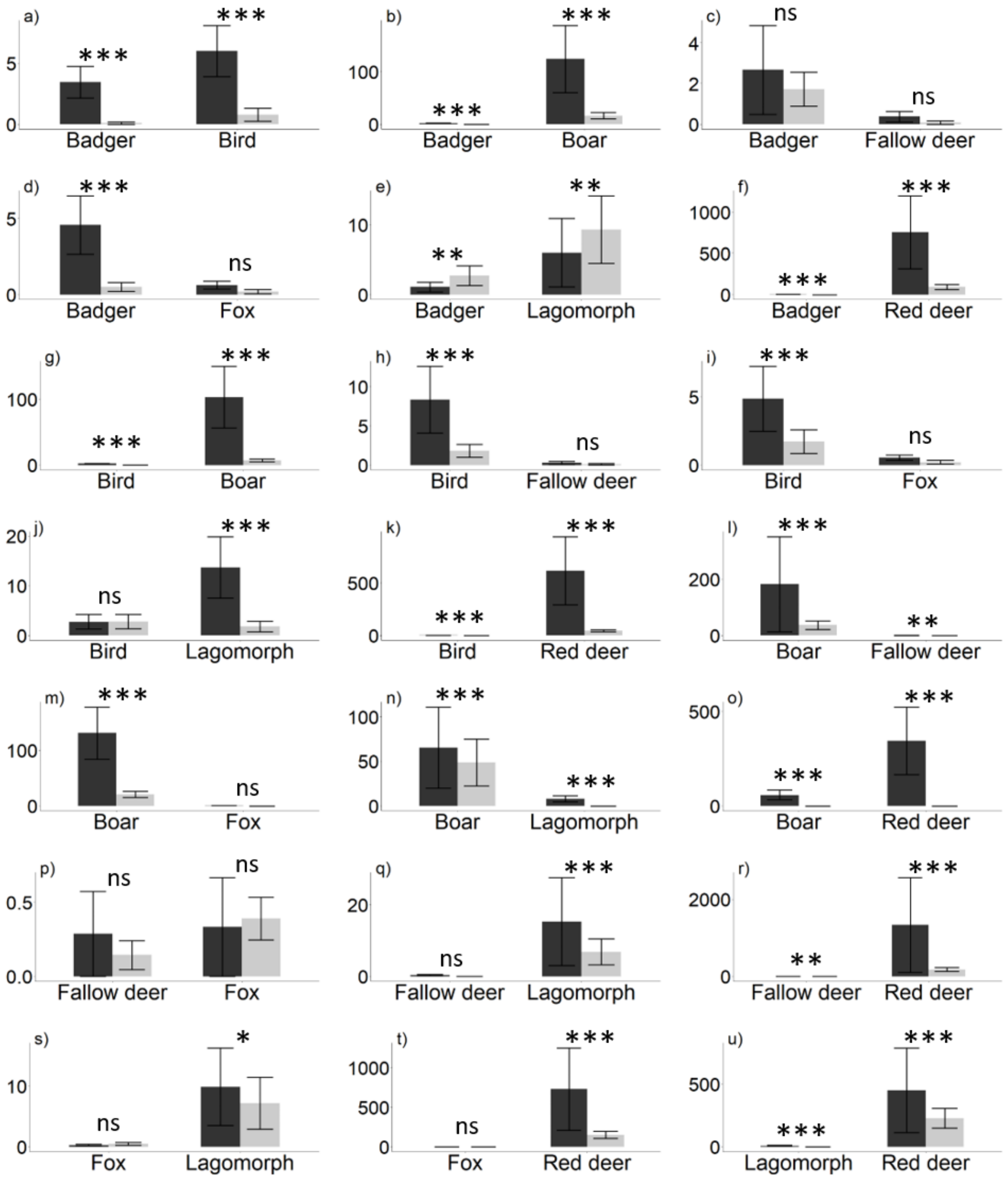

E) **Difference in *P. bourgaeana* visit patterns for frugivore pairs of species within a time window higher than 30 minutes (null model 1).** The observed values (black bars) represent the mean number of visits by a frugivore species (sp1) to individual plants visited (PV) by a second frugivore species (sp2). The expected values (grey bars) represent the mean number of visits by sp1 to plants not visited (PNV) by sp2. (* *P*<0.05, ** *P*<0.01, *** *P*<0.001).

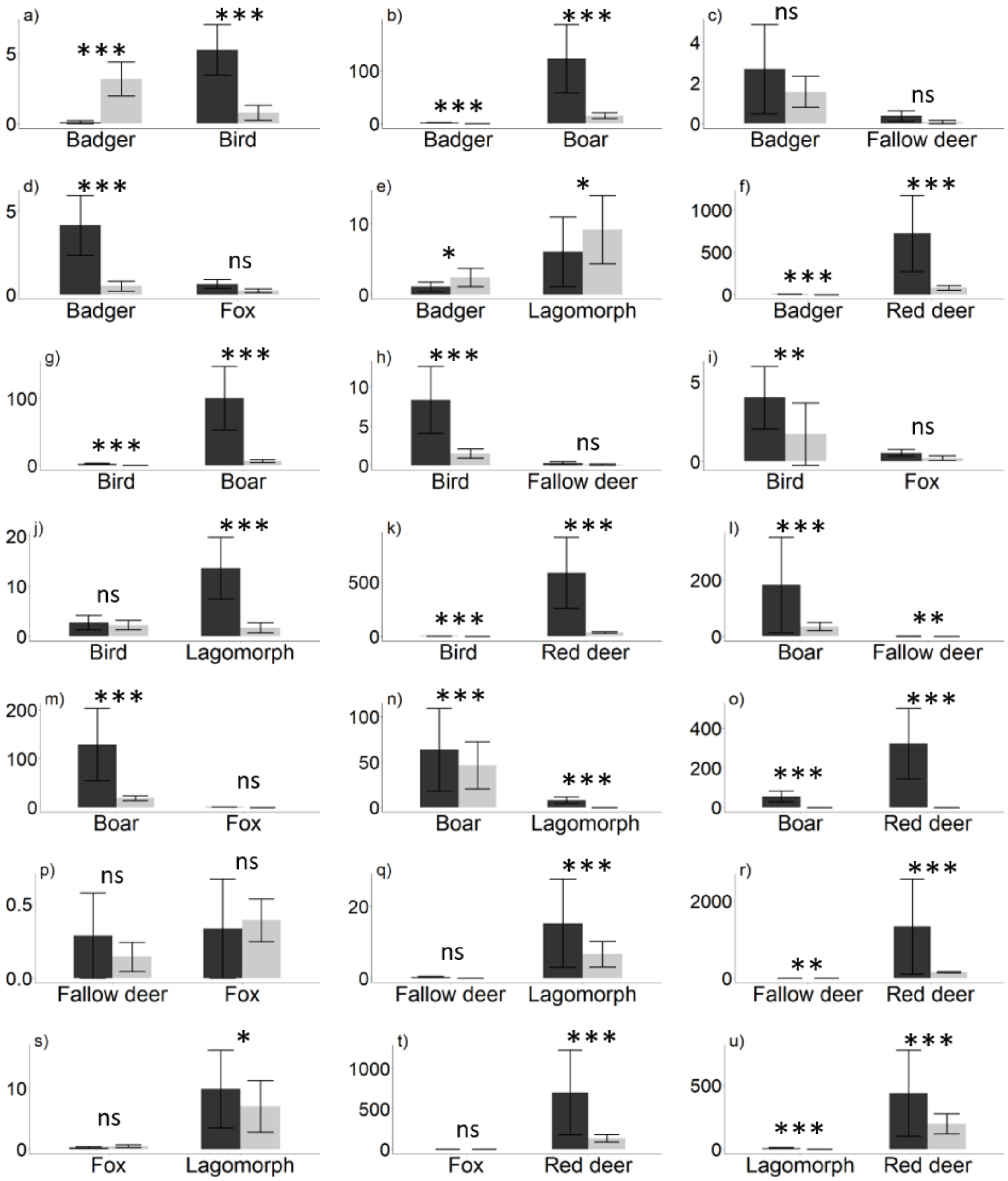

F) **Zoom of difference in *P. bourgaeana* visit patterns for frugivore pairs of species within a time window higher than 5 minutes (null model 1).** The observed values (black bars) represent the mean number of visits by a frugivore species (sp1) to individual plants visited (PV) by a second frugivore species (sp2). The expected values (grey bars) represent the mean number of visits by sp1 to plants not visited (PNV) by sp2. (* *P*<0.05, ** *P*<0.01, *** *P*<0.001).

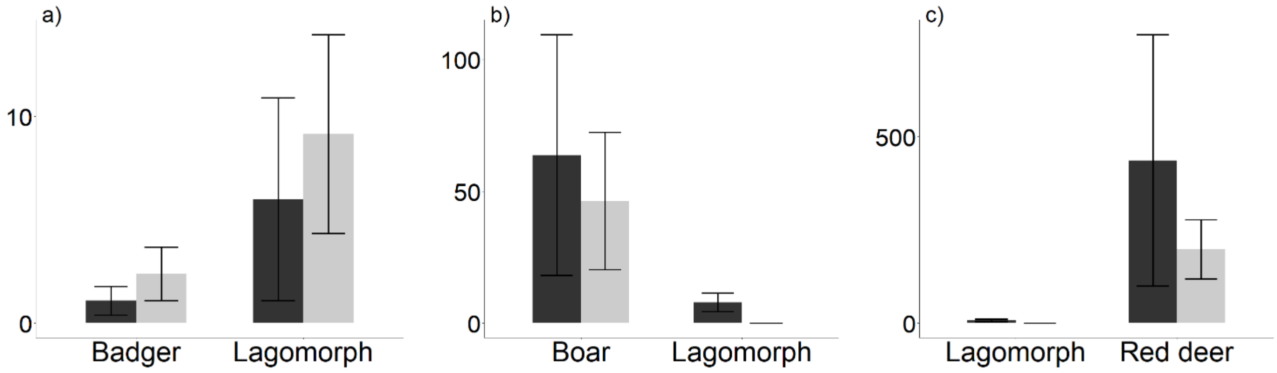

G) **Significant interactions between frugivores of *Chamaerops humilis* resulting from applying null model 2 within a time window higher than 30 minutes.** Black dots indicate the observed mean time differences (OMTD). Black lines and grey bars represent the expected 95% and 90% intervals of time elapsed between visits, respectively. (E) Interactions derived from the whole set of visit data, using *C. humilis* individuals at a minimum distance of 100 m. (F) Interactions derived from the whole set of visit data, using *C. humilis* individuals at a minimum distance of 200 m. (G) Interactions derived from data corresponding to frugivores physical interactions with *C. humilis* at a minimum distance of 100 meters. (H) Interactions derived from data corresponding to frugivores physical interactions with *C. humilis* at a minimum distance of 200 meters

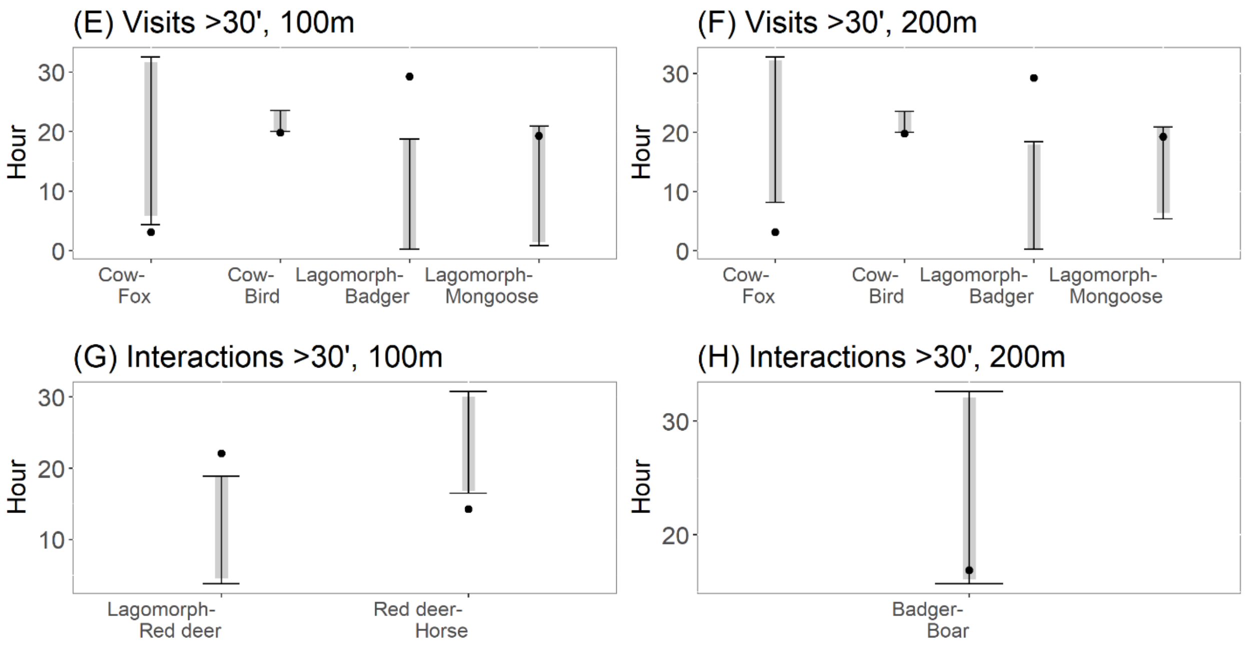

H) **Significant interactions between frugivores of *Chamaerops humilis* resulting from applying null model 3 within a time window higher than 30 minutes.** Black dots indicate the observed mean time differences (OMTD). Black lines and grey bars represent the expected 95% and 90% intervals of time elapsed between visits, respectively. (C) Interactions derived from the whole set of visit data. (D) Interactions derived from the whole set of visit data.

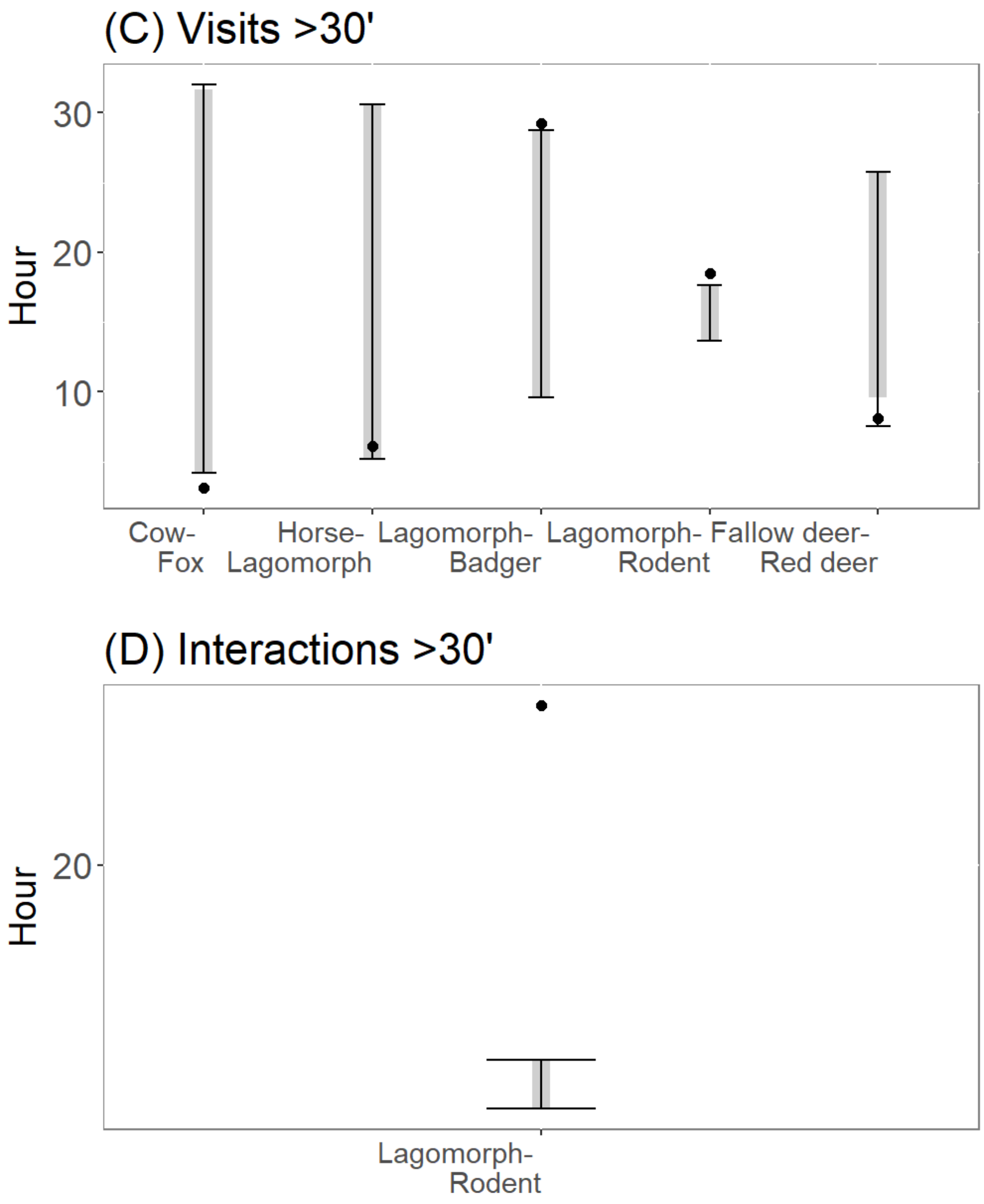

I) **Significant interactions between frugivores of *Pyrus bourgaeana* resulting from applying null model 3 within a time window higher than 30 minutes.** Black dots indicate the observed mean time differences (OMTD). Black lines and grey bars represent the expected 95% and 90% intervals of time elapsed between visits, respectively. (A) Interactions derived from the whole set of visit data.

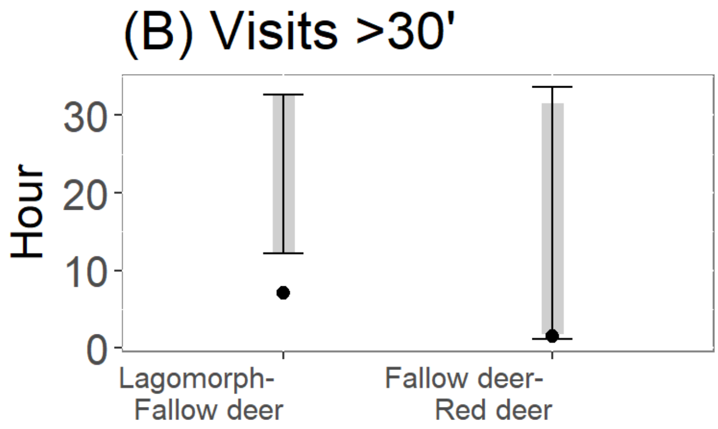

Supplement: S3 File — A) Difference in Chamaerops humilis visit patterns for frugivore pairs of species within a time window higher than 5 minutes (null model 1). B) Difference in Chamaerops humilis visit patterns for frugivore pairs of species within a time window higher than 30 minutes (null model 1). C) Zoom of difference in Chamaerops humilis visit patterns for frugivore pairs of species within a time window higher than 5 minutes (null model 1). D) Difference in P. bourgaeana visit patterns for frugivore pairs of species within a time window higher than 5 minutes (null model 1). E) Difference in P. bourgaeana visit patterns for frugivore pairs of species within a time window higher than 30 minutes (null model 1). F) Zoom of difference in P. bourgaeana visit patterns for frugivore pairs of species within a time window higher than 5 minutes (null model 1). G) Significant interactions between frugivores of Chamaerops humilis resulting from applying null model 2 within a time window higher than 30 minutes. H) Significant interactions between frugivores of Chamaerops humilis resulting from applying null model 3 within a time window higher than 30 minutes. I) Significant interactions between frugivores of Pyrus bourgaeana resulting from applying null model 3 within a time window higher than 30 minutes. (PDF) [file pone.0240614.s003.pdf]
