# Supplementary figures and images for: Interspecific interactions among functionally diverse frugivores and their outcomes for plant reproduction: A new approach based on camera-trap data and tailored null models
Source: PLoS One. 2020 Oct 16;15(10):e0240614. doi: 10.1371/journal.pone.0240614 (PMC7567357; doi:10.1371/journal.pone.0240614)

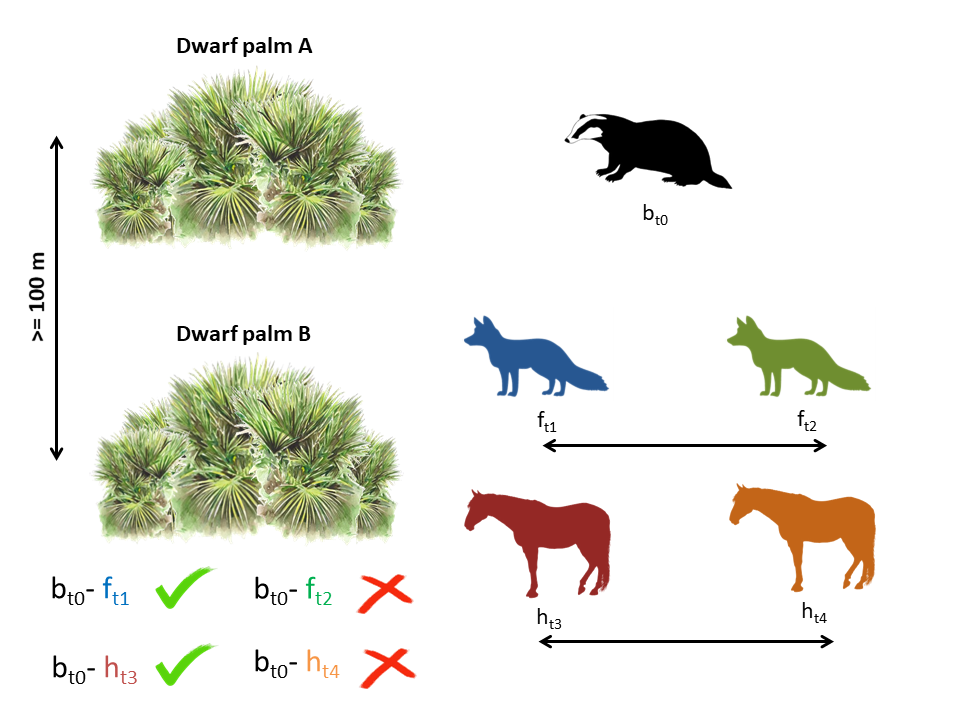

Supplement: S1 Fig — Dwarf palms (Chamaerops humilis) A and B are spatially independent individuals, in this case, separated by 100 meters from each other (the set of analysis was also run for palm trees separated 200 meters for a stricter spatial independence criterion). For this example, a badger has been recorded at dwarf palm 1 at the time bt0. For dwarf palm 2, two species have been recorded twice each: as for the first species, a fox has been recorded at time ft1 and time after, another fox has been recorded at time ft2. Whilst the second species was a horse recorded at time ht3 and after a period of time, another horse has been recorded at time ht4. To calculate expected time differences between pairs of interspecific species we have only considered the first successive visit of a different species. Therefore, for this example we have calculated time elapsed between b t0—ht3 and bt0- ft1. This scheme also applies to the Iberian pear (Pyrus bourgaeana) tree system. (TIF) [file pone.0240614.s004.tif]

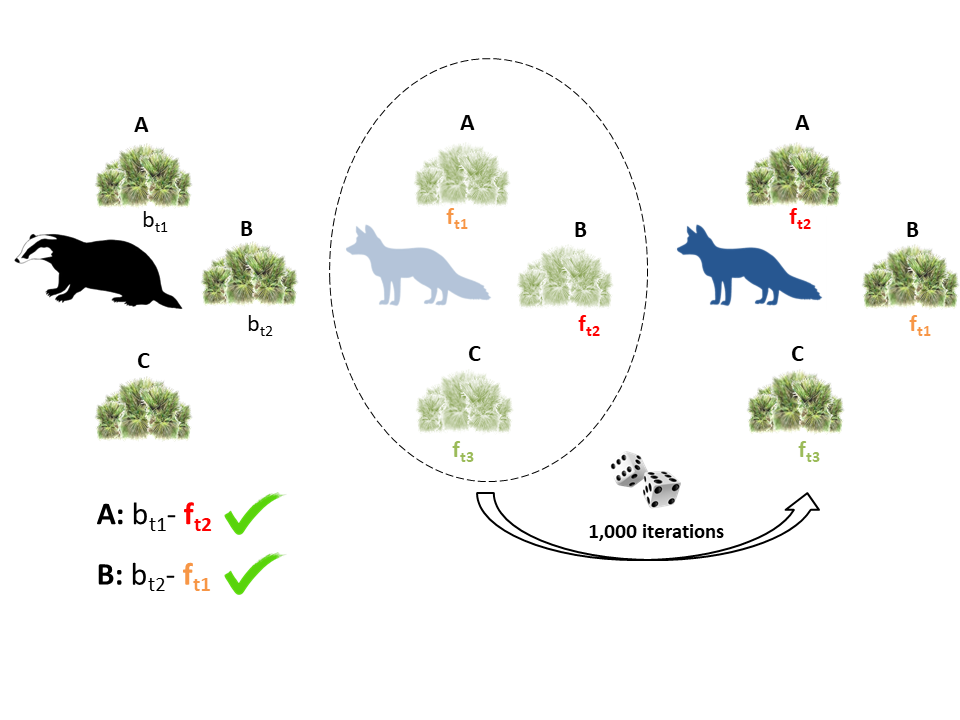

Supplement: S2 Fig — A, B and C represent Chamaerops humilis individuals at which one or both target frugivore groups have been recorded. For this example, a badger has been recorded at dwarf palms A (at time bt1) and B (at time bt2), and a red fox has been recorded at dwarf palms A (at time ft1), B (at time ft2) and C (at time ft3). To calculate expected time differences between the pair of interspecific species, we randomly assigned occurrence of frugivore groups (and the timing of the occurrences) by shuffling them 1000 times. When, by chance, both frugivore species (badger and red fox in this case) meet at the same C. humilis individual, we calculated time differences between such co-occurrence. In this example we have calculated time elapsed between bt1 and ft2 for C. humilis A and between bt2 and ft1 for C. humilis B. This scheme also applies to the Iberian pear tree (Pyrus bourgaeana) system. (TIF) [file pone.0240614.s005.tif]
