# Supplementary material for: Interspecific interactions among functionally diverse frugivores and their outcomes for plant reproduction: A new approach based on camera-trap data and tailored null models
Source: PLoS One. 2020 Oct 16;15(10):e0240614. doi: 10.1371/journal.pone.0240614 (PMC7567357; doi:10.1371/journal.pone.0240614)
Supplement: S1 Table — (PDF) [file pone.0240614.s006.pdf]

	Sampling effort days	Total photographs (5 minutes)		Total photographs (30 minutes)	
		Visits	Interactions	Visits	Interactions
C. humilis	941	1784	790	1448	568
P. bourgaeana	605	8700	5580	8227	5271
