# Supplementary material for: Interspecific interactions among functionally diverse frugivores and their outcomes for plant reproduction: A new approach based on camera-trap data and tailored null models
Source: PLoS One. 2020 Oct 16;15(10):e0240614. doi: 10.1371/journal.pone.0240614 (PMC7567357; doi:10.1371/journal.pone.0240614)
Supplement: S2 Table — Total number of visits and interactions for each frugivore functional group and plant species for both 5 and 30 minutes between successive species records. (PDF) [file pone.0240614.s007.pdf]

			**Wild ungulates**	**Domestic Ungulates**	**Carnivores**	**Pulp feeders**	**Birds**
C. humilis	Visits	5’	1260	193	135	119	74
		30’	1050	126	115	93	61
	Inter.	5’	381	37	110	54	104
		30’	327	19	92	43	84
P. bourgaeana	Visits	5’	8425	0	48	169	58
		30’	7963	0	45	167	52
	Inter.	5’	5370	0	33	146	31
		30’	5072	0	30	144	25
