# Supplementary material for: Interspecific interactions among functionally diverse frugivores and their outcomes for plant reproduction: A new approach based on camera-trap data and tailored null models
Source: PLoS One. 2020 Oct 16;15(10):e0240614. doi: 10.1371/journal.pone.0240614 (PMC7567357; doi:10.1371/journal.pone.0240614)
Supplement: S3 Table — Table 1) Chamaerops humilis 5 minutes statistical summary for null model 2. Table 2) Chamaerops humilis 30 minutes statistical summary for null model 2. Table 3) Chamaerops humilis 5 minutes statistical summary for null model 3. Table 4) Chamaerops humilis 30 minutes statistical summary for null model 3. Table 5) Pyrus bourgaeana 5- and 30-minutes statistical summary for null model 2. Table 6) Pyrus bourgaeana 5- and 30-minutes statistical summary for null model 3. (PDF) [file pone.0240614.s008.pdf]

Table 1. *Chamaerops humilis* 5 minutes statistical summary for null model 2.

Pair of species	N Observed	OMTD	N Expected	EMTD	95%ETD	Interaction
C. humilis visits (100 m)						
Cow - fox	7	3.09	12	20.52	8.31-33.56	Attraction
Cow - bird	8	19.77	11	21.20	20.21-23.58	Attraction
C. humilis visits (200 m)						
Cow - fox	7	3.09	16	22.11	8.31-34.06	Attraction
Cow - Bird	8	19.77	11	21.91	20.20-23.57	Attraction
Horse - Badger	4	18.60	4	9.57	7.39-12.40	Aversion
C. humilis interactions (100 m)						
Boar - Badger	4	7.72	9	21.82	14.97-25.97	Attraction
C. humilis interactions (200 m)						
Boar - Badger	4	7.72	9	21.82	14.97-25.97	Attraction
Fox - Horse	5	13.66	8	21.84	19.08-25.43	Attraction

Chamaerops humilis significant frugivore interactions resulting from comparison of null model 2 95%ETD and OMTD for visit and interaction data, and at least a 5 minutes period between successive same species visits. The expected mean time difference (EMTD) is also shown.

Table 2. ***Chamaerops humilis*** **30 minutes statistical summary for null model 2.**

Pair of species	N Observed	OMTD	N Expected	EMTD	95%ETD	Interaction
			C. humilis visits (100 m)			
Cow - fox	5	3.81	12	20.37	8.88-31.65	Attraction
Cow - bird	4	15.49	5	21.77	20.36-23.50	Attraction
Lagomorph - Badger	6	29.22	8	10.26	5.31-15.99	Aversion
Lagomorph - Mongoose	7	19.23	7	12.09	8.30-17.03	Aversion
			C. humilis visits (200 m)			
Cow - fox	5	3.81	9	22.25	9.06-32.14	Attraction
Cow - Bird	4	15.49	5	21.77	20.36-23.50	Attraction
Lagomorph – Badger	6	29.22	7	9.99	3.80-14.77	Aversion
Lagomorph - Mongoose	7	19.23	6	14.07	12.32-18.98	Aversion
			C. humilis interactions (100 m)			
Lagomorph - Red deer	7	22.11	20	13.94	8.02-23.56	Aversion
Red deer - Horse	4	14.22	21	16.42	8.37-24.55	Attraction
			C. humilis interactions (200 m)			
Badger - Boar	4	16.87	14	17.78	6.32-27.83	Attraction

Chamaerops humilis significant frugivore interactions resulting from comparison of null model 2 95%ETD and OMTD for visit and interaction data, and at least a 30 minutes period between successive same species visits. The expected mean time difference (EMTD) is also shown.

Table 3. ***Chamaerops humilis*** **5 minutes statistical summary for null model 3.**

**Pair of species**	**N Observed**	**OMTD**	**N Expected**	**EMTD**	**95%ETD**	**Interaction**
C. humilis visits						
Cow - Fox	**7**	3.09	25	17.59	6.45-32.02	Attraction
Horse - Lagomorph	13	6.10	14	19.97	5.90-30.58	Attraction
Lagomorph - Badger	6	29.22	127	20.86	20.12-22.62	Aversion
Lagomorph - Rodent	10	18.51	150	16.10	14.8-17.78	Aversion
C. humilis interactions						
Lagomorph - Rodent	5	23.43	69	17.55	17.63-17.78	Aversion
Bird - Boar	13	7.71	69	23.49	14.9-33.11	Attraction

Chamaerops humilis significant frugivore interactions resulting from comparison of null model 3 95%ETD and OMTD, for visit and interaction data, and at least a 5 minutes period between successive same species visits. The expected mean time difference (EMTD) is also shown.

Table 4. ***Chamaerops humilis*** **30 minutes statistical summary for null model 3.**

Pair of species	N Observed	OMTD	N Expected	EMTD	95%ETD	Interaction
C. humilis visits						
Cow - Fox	7	3.09	32	17.06	8.47-25.67	Attraction
Horse - Lagomorph	13	6.10	11	17.25	6.47-29.91	Attraction
Lagomorph - Badger	6	29.22	129	20.70	20.11-22.61	Aversion
Lagomorph - Rodent	10	18.51	91	15.67	14.23-17.63	Aversion
Fallow deer - Red deer	9	8.11	41	18.76	14.88-23.00	Attraction
C. humilis interactions						
Lagomorph - Rodent	4	21.94	63	17.25	17.03-17.63	Aversion

Chamaerops humilis significant frugivore interactions resulting from comparison of null model 3 95%ETD and OMTD, for visit and interaction data, and at least a 30 minutes period between successive same species visits. The expected mean time difference (EMTD) is also shown.

Table 5. ***Pyrus bourgaeana*** **5 and 30 minutes statistical summary for null model 2.**

Pair of species	N Observed	OMTD	N Expected	EMTD	95%ETD	Interaction
P. bourgaeana visits 5’ and 30’						
Fallow deer - Red deer	4	1.56	5	11.42	2.62-17.10	Attraction

Pyrus bourgaeana significant frugivore interactions resulting from comparison of null model 2 95%ETD and OMTD, for visit data, and at least 5- and 30-minutes period between successive same species visits. The expected mean time difference (EMTD) is also shown.

Table 6. ***Pyrus bourgaeana*** **5 and 30 minutes statistical summary for null model 3.**

Pair of species	N Observed	OMTD	N Expected	EMTD	95%ETD	Interaction
			P. bourgaeana visits 5’			
Red deer - Fox	7	18.58	11	25.31	30.44-33.76	Attraction
Lagomorph - Fallow deer	4	7.11	8	10.72	12.63-13.82	Attraction
Fallow deer - Red deer	4	1.56	21	17.96	22.6526.62	Attraction
			P. bourgaeana visits 30’			
Lagomorph - Fallow deer	4	7.11	12	17.02	16.07-32.69	Attraction
Fallow deer - Red deer	4	1.56	22	18.91	25.34-31.55	Attraction

Pyrus bourgaeana significant frugivore interactions resulting from comparison of null model 3 95%ETD and OMTD, for visit data, and at least 5 and 30 minutes period between successive same species visits. The expected mean time difference (EMTD) is also shown.
