# Supplementary material for: Interspecific interactions among functionally diverse frugivores and their outcomes for plant reproduction: A new approach based on camera-trap data and tailored null models
Source: PLoS One. 2020 Oct 16;15(10):e0240614. doi: 10.1371/journal.pone.0240614 (PMC7567357; doi:10.1371/journal.pone.0240614)
Supplement: Attachment — Submitted filename: Selwyn et al. Response to Reviewers.pdf [file pone.0240614.s009.pdf]

INSTITUTO SUPERIOR DE AGRONOMIA
Universidade de Lisboa
Hinc Patriam Sustinet

Miriam Selwyn
Centro de Ecologia Aplicada
“Prof. Baeta Neves”
Tapada da Ajuda
1349-017 Lisboa, PORTUGAL

phone no. +34603639427
e-mail: mimselwyn@gmail.com

Dr. Joerg Heber
Editor-in-Chief:
PLOS ONE
Public Library of Science:
San Francisco, CA, US

Lisbon, August 23th 2020

Dear Editor,

Many thanks for the opportunity to revise our manuscript entitled “Interspecific interactions among functionally diverse frugivores and their outcomes for plant reproduction: a new approach based on camera-trap data and tailored null models” (PONE-D-20-17034).

We are also thankful to the Associate Editor Pedro G. Blendinger and the two reviewers for their helpful and thoughtful comments and suggestions. We have rewritten the manuscript following closely both reviewers’ concerns as well as the Editor’s comments. The three major changes done as response to these suggestions are:

1. As suggested by reviewers #1 and #2, we have improved the illustration of the study system, relations between species, and the functional groups.

2. Following the reviewer #2 and the Editor’s suggestions, we have set up a leaner version of the manuscript, significantly reducing the Results section.

3. We expanded the Discussion and Conclusion sections and including a number of new references from research done elsewhere as proposed by the editor and both reviewers.

Please, find attached our responses (R. -) to the comments and suggestions (Ci. -). All answers are referred to the revised version of the manuscript that includes “track changes”. Thank you again for your kind assistance and the time devoted to our manuscript.

Yours sincerely,

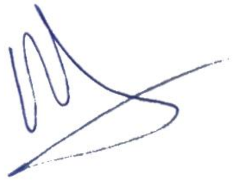

Responses to Additional Editor Comments:

C1. - Consider modifying the title, so that it is not so general and fully reflects the main objectives of the study; see also the comment of Reviewer # 1.

R. – Many thanks for the suggestion. We have modified the title, for it to be more specific, to: "*Interspecific interactions among functionally diverse frugivores and their outcomes for plant reproduction: a new approach based on camera-trap data and tailored null models*".

C2. - Avoid making lack of knowledge a strong point of the study (e.g. lines 53-54, 59-62, 70-71). Instead, highlight why this new knowledge is important, what its contribution is, and how it enables progress on ecological theory.

R. – The contributions to other methodological frameworks and implications on ecological processes have been highlighted throughout the abstract (lines 46-49), discussion and conclusion (lines 696-705 and 706-711) of the manuscript.

For example:

L46-49: "*Our new analytical approach provides a widely applicable framework for further studies on multispecies interactions in different systems beyond plant-frugivore interactions, including plant-pollinator interactions, the exploitation of plants by herbivores, and the use of carcasses by vertebrate scavengers*".

L696-705: "*This new approach may provide advantages and complementarities to ongoing research and methodologies for multispecies interactions such as multilayer networks or spatially explicit agent-based simulation modeling [19-20, 89]. For instances, by relaying on data from camera-trapping, our approach can be applied to uncover interspecific interactions among understudied nocturnal or otherwise elusive animals such as many mammals and other secretive vertebrates. Our analytical approach enabled us to evaluate the specific hypothesis and predictions that we had projected for our particular study system and would hardly have been able to achieve using other similar approaches (see 26-28) that impose a number of requirements and assumptions that did not always fit our specific system".*

L706-711: *"This study may also foster a greater understanding of the implications of multispecies plant-animal interactions on ecosystem function and restoration by means of direct and indirect interaction pathways that influence seed dispersal and recruitment in disturbed areas [90]. It can also enrich a great potential of interaction network researches predicting indirect novel interactions or changes in functional network structures under global changing conditions (e.g. land use changes, human disturbance and climate change) [35]".*

C3. - Lines 88-96. Significantly reduce the species description in the Introduction. For the reader unfamiliar with the species on your study site, it is more important to know the functions of the species than their identity. Reviewer's # 1 recommendation on citing evidence of competitive interactions in the system should be included in Methods.

R. – This is a good point. Thank you! We have significantly reduced the species description in the introduction section and have summarized a reduced description of the species functions instead (lines 103-108:

"*This diverse assemblage of frugivores that consume C. humilis and P. bourgaeana's fruits can be grouped into five functional frugivore groups: carnivores, wild ungulates, domestic ungulates, pulp feeders (i.e. lagomorphs and rodents) and birds which may act as seed disperses or seed predators [18, 29-33]; thus, providing a wide spectrum of antagonistic and mutualistic interactions among multiple species across trophic levels [32, 34-35].*").

Also, we have stated in the Methods a detailed description of the different frugivore species (lines 174-212:

"In Doñana National Park, *C. humilis* and *P. bourgaeana*'s seed dispersal is mainly accomplished by medium-size carnivores such as Eurasian badgers (*Meles meles*) and red foxes (*Vulpes vulpes*) [28-29]. These two carnivores intensively prey on lagomorphs (*Oryctolagus cuniculus*) and up to five rodent species (mostly *Apodemus sylvaticus* and *Mus spretus*) [32] that feed on *C. humilis* and *P. bourgaeana*'s pulp and seeds [29-30, 33]. Lagomorphs and rodents act mostly as pulp feeders, although may occasionally act as short-distance seed dispersers for *C. humilis* [29]. Whereas for *P. bourgaeana*, lagomorphs act mostly as pulp feeders and may occasionally disperse some seeds at short distances, rodents damage all seeds eaten [18, 30]. Fruit predators such as red (*Cervus elaphus*) and fallow deer (*Dama dama*) grind all ingested seeds [29-30], although can disperse viable seeds of *C. humilis* by regurgitation [31]. Wild boars (*Sus scrofa*) act mostly as seed predators though may occasionally disperse some viable

seeds of both species [30, P.J. Garrote *unpublished data*]. Free-ranging cows and horses are abundant in some areas of Doñana and are known to consume and usually predate both *C. humilis* and *P. bourgaeana* seeds. Large mammals (e.g. deer, cows) seem to be better adapted in their foraging, either by breaking many of the defensive needle-like spines in fruiting *C. humilis* or by plucking *P. bourgaeana*'s fruits still attached to the branches (Authors *personal observations*, see S1 File A). Hence, ungulates remove many fruits of both plant species and often leave some of them whole or partially consumed around mother plants (Authors *unpublished data*). These left fruits are more easily accessible to medium-sized frugivores which hardly cope with the palm spines and cannot reach the fruits attached to *P. bourgaeana* branches. Therefore, we predict that the foraging of smaller frugivores could be facilitated by larger ones [e.g., 18, 53-55]. Also, birds such as azure-winged magpie *Cyanopica cyanus* and blackcap *Silvia atricapilla* consume *P. bourgaeana*'s fruits mostly acting as pulp-predators rather than seed dispersers [30]. As a result, this diverse assemblage of frugivores can be grouped into the above-mentioned five functional groups (carnivores, wild ungulates, domestic ungulates, pulp feeders and birds).")

C4. - Line 132. Please inform on the polydrupe size.

R. – Done. See lines 163-165: "*Its fruits are 'polydrupes' (usually 1-3 drupes, which vary from 1-3 cm in diameter [49]) attached to infrutescences of up to 30 cm long (7-120 fruits per infructescence) and usually located at ~10-30 cm above the ground level*".

C5. - Lines 178-191 should be condensed but be careful not to omit relevant information.

R. – Thanks for the suggestion. We have moved this information to the supplementary material (S1 Table, see lines 239-240).

C6. - Line 217. Use italics for "expected time difference".

R. – Done. See line 282.

C7. - I do not understand why you show plant crop size variation and frugivore visitation as a separate result. This is confusing and contributes to unnecessarily complexing a manuscript that is already quite complex. Although it is an interesting topic, it is not one of your objectives. In this study, crop size should be more a source of variation to be controlled than a main effect to be evaluated.

R. – The editor is right. For both plant species, we have moved the information related to crop size variation to null model's 1 section (see lines 321-333 for *C. humilis* and lines 487-501 for *P. bourgaeana*). To evaluate whether crop size affected the outcome of our analyses, we added in our regression models "crop size" as an environmental co-variable, and this did not alter the output of the frugivore species interactions. Therefore, for the sake of conciseness, we have omitted these new analyses from the manuscript. Nevertheless, we now mention in the Methods section that patterns derived from null model 1 are consistent when crop size is added a covariate. See response to reviewer's 2, comment 2 (C2) for complete information**.**

C8. - The potential influence on visit frequency of the fruiting neighborhood does not appear to have been considered. This may be an important limitation of the study; I would expect to see some consideration in this regard in the Discussion. Along the same line, see the comments of both reviewers about the limitations of a non-spatially explicit design in interpreting the results.

R. – Indeed, the potential visit frequency of fruiting neighbor plants may be affecting our results and that is why we first applied null model 1. Specially, null model 1 was applied to test whether or not the different frugivore species used the same fruiting individuals. Once we revealed that, in general, most species used the same fruiting plants, and therefore are exposed to the same environmental conditions (including neighbor's crop size and visit frequency) we then tested their temporal relation applying null models 2 and 3. In contrast to null model 1, the results of these two analyses are not potentially influenced by changes in environmental conditions among individual fruiting plants since the pool of plants considered is the same for each pair of frugivore species considered and we evaluated changes in the timing of frugivore visit for a given individual plant.

C9. - Results are hard to follow, and even more difficult to interpret without falling into successive ad-hoc explanations. I

recommend reducing them, keeping only those most closely related to the main objective and the proposed conceptual framework. In the context of plant-animal interactions in which you framed the study (e.g. lines 76-78), interactions are the most important, not visits. On the other hand, it appears that a 5 min interval between visits may better reflect an effect of responses to previous visitors than a 30 min interval. Therefore, I suggest that you only focus the results in number of interaction using the 5 min as a reference. The other results can be omitted entirely, or can be reduced to the minimum possible in the main text, referring to the supplementary material to see the complete complementary analyzes. This includes the corresponding figures and tables.

R. – Many thanks for the suggestion. We have significantly reduced the Results section for both *C. humilis* and *P. bourgaena*. The major changes that are found in the revised version of the manuscript are:

Null model 1 - We have merged "Overall patterns" and "null model 1" sections (see lines 321-333 for *C. humilis* and lines 487-501 for *P. bourgaeana*. Also, we have synthesized this section keeping the most important paired interactions (see new Figures 2 and 5) and omitted 30-minute results, as they were consistent with 5-minute ones (moving the complete outcome to supplementary materials).

Null model 2 - Tables 1, 2 have been moved to Supplementary Material (See S3 Table).

Null model 3 – Tables 3, 4 and 5 have also been moved to Supplementary Material (See S3 Table).

Regarding results corresponding to a time window higher than 30 minutes, we have completely omitted this outcome from the Result section and have moved this information to the Supplementary Materials (See S3 File) and lines 553-559 of the manuscript:

"*Very similar patterns were found when applying the 3 null models for visit and interaction data within a time window higher than 30 minutes. Thus, regardless of the criteria used to select the dataset, for most cases, frugivore pairs used the same C. humilis and P. bourgaeana fruiting individuals. Most of the resulting interactions were consistent when restricting the analysis to a narrower time window (30 minutes). Further*

details on the complete outcome, figures and statistical summaries (for both 5 and 30 minutes) can be found in S3 File and S3 Table, respectively."

Regarding the "visit results", we believe that visits and interactions are closely linked. For example, the mere presence of a visitor (e.g. predator), independently of whether is eating fruits or not, may affect the subsequent visitor's (e.g. prey) foraging pattern. Therefore, as above, we have condensed as much as possible results and figures and tables, presenting only those interactions that are considered most important. We however have kept the most representative results concerning frugivore visitation.

C10. - The Discussion is somewhat poor and does not take advantage of the potential of the results. For example, it could be greatly enriched by focusing more on functional interpretation and the implications for community organization.

R. – Many thanks for the suggestion. In line with reviewers' 1 and 2 comments we have included more discussion about functional interpretations and community organization.

For example, we now mention that:

"*Other environmental conditions derived from the micro (e.g. plant's location, herbaceous cover, distance to shrub and light availability) and meso habitat (e.g. elevation and type of forest cover) related to each individual plant as well as each animal's niche differences, that allow the coexistence of consumer species, could also exert an effect on the spatial pattern of frugivore visitation [62, 71]. In our system, most of the species (carnivores and ungulates) show high mobility, being able to travel several kilometers within the same day. Therefore, and given the relatively small spatial scale of our study sites, it can be assumed that different species of frugivores had access to all fruiting plants. This scenario however differs for rodents and lagomorphs since they are less mobile; thus, a few cases of spatial avoidance by small mammals identified (null model 1) should be considered with caution"* (lines 612-621).

"*Facilitative interactions in the acquisition of food are highly prevalent among many terrestrial vertebrates and have critical importance in structuring and function of many communities [53-55, 76-77]. In contrast with Carreira et al.'s [27] findings, we detected that medium-sized frugivores tended to be temporally attracted by larger ungulates. (…) Our study shows how this functionally diverse assemblage of frugivores leads to interspecific facilitation in foraging, a widespread pattern in other temperate and tropical habitats [55, 76-77]. Positive interactions may be an important mechanism linking high*

diversity to high productivity under stressful environmental conditions [54, 78]" (lines 635-655).

"*As predicted, seed predators and pulp feeders can alter the interaction frequency between fleshy-fruited plants and their mutualistic and antagonistic animal associates [18]. In the first case, ungulate seed predators increased the interaction frequency with legitimate seed dispersers, for both C. humilis and P. bourgaeana yielding thus an indirect positive effect on seed survival (Fig. 7A). Secondly, pulp feeders, that often lessen long-distance dispersal [52], decreased C. humilis' foraging by ungulate seed predators, enhancing thus seed survival and fruit removal by mutualistic animal associates (Fig. 7B)*" (lines 664-670).

"*Therefore, both, mutualistic and antagonistic partners that shape the interaction outcomes among individual plants lead to contrasting seed dispersal success [18, 70], appearing to shape a complex web of direct and indirect effects which's net effect is most likely dependent on the community context [52]. This outcome may be strongly dependent on initial population densities that would most likely alter the dynamics of the system, thus, the net effect of dispersal success, among other factors, will probably be a result of the abundance of different functional guild of frugivores (legitimate seed dispersers, pulp feeders and seed predators) [87-89]*." (lines 680-686).

C11. - Figure 8 should move to results.

R. – Many thanks for the suggestion. We have moved Figure 7 (former Figure 8) to the Result section (see lines 579-582 for the Figure's description:

"***Fig 7. Effects on C. humilis and P. bourgaeana's reproductive success.*** *General direct and indirect interaction pathways between pairs of frugivore species in relation to Chamaerops humilis and Pyrus bourgaeana reproductive success based on interaction patterns obtained from null models 2 and 3"*).

Also, we have added information about the outcome of summing up the results derived from the application of the null models in lines 565-577:

"*As a result of summing up the outcomes of null models 2 and 3, we assessed both direct and indirect interaction pathways among C. humilis and P. bourgaeana frugivores' in relation to these plants reproductive success. For instance, we found that large ungulates such cows or red deer, had a direct (mostly) negative effect on seed dispersal as they grind most ingested seeds (Fig 7A). However, cows had a direct positive effect*

for legitimate seed dispersers, such as red foxes which were attracted to this large ungulate and thus, lead to an indirect positive effect on C. humilis' reproductive success (Fig 7A). Our results also showed a similar situation for the interactions between red deer, red foxes, and P. bourgaeana (Fig 7A). Finally, our study also disclosed how, for instance, lagomorphs which mainly had a direct negative effect on C. humilis reproductive success acting as pulp feeders, also had a direct negative effect on seed predators such as red deer. Thus, these direct interspecific interactions lead to an indirect positive effect on C. humilis reproductive success (Fig 7B)".

C12. - Finally, there is a marked tendency to self-refer, and not always because they are the only references available. It is important that you smooth this trend significantly.

R. – It may appear to exist a self-refer tendency but this is due to the broad knowledge of the co-authors on the study area and species within it. Even so, please, find bellow a compilation of the added references:

- Palomares F, Ferreras P, Fedriani JM, Delibes M (1996). Spatial relationships between Iberian lynx and other carnivores in an area of south-western Spain. Journal of Applied Ecology, 5-13

- Barasona JA, Latham MC, Acevedo P, Armenteros JA, Latham ADM, Gortazar C, et al. (2014). Spatiotemporal interactions between wild boar and cattle: implications for cross-species disease transmission. Veterinary research, 45, 122.
- Triguero-Ocaña R, Barasona JA, Carro F, Soriguer RC, Vicente J, Acevedo P (2019). Spatio-temporal trends in the frequency of interspecific interactions between domestic and wild ungulates from Mediterranean Spain. PloS one, 14, e0211216
- Valverde JA (1967) Estructura de una comunidad de vertebrados terrestres. Estación Biológica de Doñana. Monografia, 1.
- Kissling WD, & Schleuning M. (2015) Multispecies interactions across trophic levels at macroscales: retrospective and future directions. Ecography, *38*(4), 346-357.
- Ackerly DD, & Cornwell WK (2007) A trait-based approach to community assembly: partitioning of species trait values into within-and among-community components. Ecology letters, 10(2), 135-145.
- Finke DL, & Snyder WE (2008) Niche partitioning increases resource exploitation by diverse communities. Science, 321(5895), 1488-1490.

- Chase JM, & MA Leibold (2003) Ecological niches. Linking classical and contemporary approaches. University of Chicago Press.
- Valdés-Castrillón B, Talavera-Lozano S, & Fernández-Galiano FE (1987) Flora vascular de Andalucía Occidental, 3 volúmenes. Ketres Editora SA, Barcelona, España.
- Hernandez A (2008) Cherry removal by seed-dispersing mammals: mutualism through commensal association with frugivorous birds. Polish Journal of Ecology, 56(1), 127.
- Owen-Smith RN (2002) Adaptive herbivore ecology: from resources to populations in variable environments. Cambridge University Press.
- Severo-Neto F, Yule TS, & Costa-Pereira R (2011) Parakeets as facilitators of fish frugivory in the Southern Pantanal. Atualidades Ornitológicas, 169, 15.
- Perry RW, & Thill RE (1999) Estimating mast production: an evaluation of visual surveys and comparison with seed traps using white oaks. Southern Journal of Applied Forestry, 23(3), 164-169.
- Koenig WD, Knops JM, Carmen WJ, Stanback MT, & Mumme RL (1994) Estimating acorn crops using visual surveys*. Canadian Journal of Forest Research, 24*(10), 2105-2112.
- Nakajima A, Masaki T, Koike S, Yamazaki K, & Kaji K (2015) Estimation of tree crop size across multiple taxa: generalization of a visual survey method. Open Journal of Forestry, 5(07), 651.
- Ameztegui A, & Coll L (2015) Herbivory and seedling establishment in Pyrenean forests: influence of micro-and meso-habitat factors on browsing pressure. Forest Ecology and Management, *342*, 103-111.
- Bolker BM, Brooks ME, Clark CJ, Geange SW, Poulsen JR, Stevens MHH, & White JSS (2009) Generalized linear mixed models: a practical guide for ecology and evolution. Trends in ecology & evolution, 24(3), 127-135.
- Anderson S (2012) A quick introduction to plyr.
- Mailund T (2019) Manipulating Data Frames: dplyr. In R Data Science Quick Reference (pp. 109-160). Apress, Berkeley, CA.
- Grolemund G, & Wickham H (2011) Dates and times made easy with lubridate. Journal of Statistical Software, 40, 1-25.
- García D, Zamora R, & Amico GC (2011) The spatial scale of plant–animal interactions: effects of resource availability and habitat structure. Ecological Monographs, 81(1), 103-121.
- García D, & Martínez D (2012) Species richness matters for the quality of ecosystem services: a test using seed dispersal by frugivorous birds. Proceedings of the Royal Society B: Biological Sciences, 279(1740), 3106-3113.
- Quitián M, Santillán V, Espinosa CI, Homeier J, Böhning-Gaese K, Schleuning M, & Neuschulz EL (2018) Elevation-dependent effects of forest fragmentation

on plant–bird interaction networks in the tropical Andes. Ecography, *41*(9), 1497-1506.
- Schaefer HM, & Ruxton GD (2011) Plant-animal communication. OUP Oxford.
- Nathaniel Holland J, & DeAngelis DL (2009) Consumer-resource theory predicts dynamic transitions between outcomes of interspecific interactions. Ecology Letters, 12(12), 1357-1366.
- Wang, Y., DeAngelis, D.L. & Holland, J.N. (2012) Uni-directional interaction and plant–pollinator–robber coexistence. *Bulletin of Mathematical Biology*, **74**, 2142–2164.
- Fedriani JM, D Ayllón, T Wiegand, and V. Grimm (2020) Intertwined effects of defaunation, increased tree mortality, and density compensation on seed dispersal. Ecography, DOI: 10.1111/ecog.05047.
- Buckley YM, Anderson S, Catterall CP, Corlett RT, Engel T, Gosper CR, et al., (2006) Management of plant invasions mediated by frugivore interactions. Journal of Applied Ecology, 43(5), 848-857.
- Waser NM, & Ollerton J (2006) Plant-pollinator interactions: from specialization to generalization. University of Chicago Press.
- Benbow ME, Tomberlin JK, & Tarone AM (2015) Carrion ecology, evolution, and their applications. CRC press.

Responses to Reviewer #1

C1. - This manuscript addresses in detail how animal functional groups interact with two fruiting plants, and how some functional groups influence the exploitation of those fruiting plants on a temporal and spatial scale in the Doñana National Park, SW of the Iberian Peninsula. More importantly, the authors provide new a new approach by combining camera trapping and null models to figure out those interactions. It appears that some functional groups of frugivores have negative or positive effects on the exploitation of fruit plants by others and this may have consequences on the reproductive success of fruiting plants. The study opens a new door for studying plant-animal interaction and seed dispersal at the community level to understand the functioning of ecosystems. I was particularly interested in the level of details given in the result section.

However, some aspects need clarification in the abstract, introduction, methods, and discussion.

R. – Many thanks to this reviewer for his positive appreciation. Along the revision of the manuscript we have included or re-written information following the reviewer's comments and suggestions to clarify some aspects of the manuscript for the reader to have a better understanding.

Abstract

C2. - Lines 16-20: This sentence refers to lines 73-75. However here the word "combination" that makes the approach new according to me is lacking.

R. – Many thanks for the suggestion. We believe the reviewer is right and have included the word "combines" in this sentence (see line 24):

"*To do so, we developed a new analytical approach that combines camera trap data (spatial location, visitor species, date and time, activity) and tailored null models that allowed us to infer spatial-temporal interactions (attraction, avoidance or indifference) between pairs of frugivore species.*"

C3. - Lines 30-32: This objective seems to be part of the research. So I would suggest considering revising the third sentence of the abstract (lines 15-16) to include this aspect and give here the outcome of this analysis, instead of stating the objective.

R. – The reviewer is right. We deleted the objective described in lines 30-32 and we have re-written this sentence of the abstract including this information (lines 22-23):

"*Then, we inferred how the revealed direct interspecific interactions among frugivores could lead to indirect (positive or negative) effects on reproductive success of fleshy fruited plants.*".

Also, we have included the outcome of this analysis in lines 39-44:

"*Finally, our results allowed us to identify direct interaction pathways, that revealed how the mutualistic and antagonistic relations between animal associates derived into indirect effects on both plants seed dispersal success. For instance, we found that large-sized seed predators (e.g. ungulates) had a direct positive effect on the likelihood of visits by legitimate seed dispersers (e.g. red foxes) to both fleshy fruited plants. Then, seed predators showed an indirect positive effect on the plants' reproductive success.".*

C4. - Line 35 (carcasses): Maybe "fruit remains", it is generally called "feeding remains"

R. – Indeed, this study may also be applied to the use of "fruit remains" by some species. However, our intention here was not to describe the use of fruit remains by frugivores but a completely different ecological interaction such as the use of animal remains (carcasses) by their consumers (scavengers):

L46-49: "*Our new analytical approach provides a widely applicable framework for further studies on multispecies interactions in different systems beyond plant-frugivore interactions, including plant-pollinator interactions, the exploitation of plants by herbivores, and the use of carcasses by vertebrate scavengers*".

Introduction

C5. - Lines 51-53: Please can the author provide some examples of studies of different types of interactions taking place in the same habitat?

R. – Done, see line 65. References included in the text:

15. Palomares F, Ferreras P, Fedriani JM, Delibes M (1996). Spatial relationships between Iberian lynx and other carnivores in an area of south-western Spain. Journal of Applied Ecology, 5-13.
16. Barasona JA, Latham MC, Acevedo P, Armenteros JA, Latham ADM, Gortazar C, et al. (2014). Spatiotemporal interactions between wild boar and cattle: implications for cross-species disease transmission. Veterinary research, *45*, 122.
17. Triguero-Ocaña R, Barasona JA, Carro F, Soriguer RC, Vicente J, Acevedo P (2019). Spatio-temporal trends in the frequency of interspecific interactions between domestic and wild ungulates from Mediterranean Spain. PloS one, *14*, e0211216.

C6. - Lines 78-82: a) the authors only treated this in part in the abstract. Please provide information on the outcome of the first step in the abstract.

R. – a) We are sorry if this has led to misunderstanding. The two steps refer to the three null models explained in the "analytical approach" section. Therefore: Step one refers to null model 1 and step two refers to null models 2 and 3 as both are based in the same type of data. The information about the outcome of the first step can be found in lines 33-35 of the abstract:

"*Our first step showed, for instance, that some prey frugivore species (e.g. lagomorphs) tend to avoid those C. humilis individuals that were most visited by their predators (e.g. red foxes).)*".

b) Please consider revising the tense of the verbs in both sentences. The first is in the present tense and the second in the past tense.

R. – b) Done! Thank you.

C7. - Lines 82-84: a) The way this research question is stated (using "how") directly means that we already acknowledge that there would be an effect. I would suggest saying, "we inferred the consequence of these direct responses on the plants", instead of "how they would affect".

R. – a) Done! Many thanks for the suggestion. See lines 96-99: "*Finally, we inferred the indirect consequences of these direct frugivore responses (attractive or aversive) on the plant's reproductive success [e.g., 18]*".

b) This question seems to form an integral part of the present research, as we can see in the second sentence of the abstract. Although it is mention in the abstract, it is not clear from the title of the manuscript that the interaction between the frugivores and plants will be analyzed.

b) Thank you. Indeed, this is an important argument in our study. Therefore, we have changed the title to: "*Interspecific interactions among functionally diverse frugivores and their outcomes for plant reproduction: a new approach based on camera-trap data and tailored null models*"

C8. - Line 85: Please consider revising the tense. Some other mistakes may be found elsewhere.

R. – Done. Thanks. (Lines 92-95).

C9. - Lines 96-99: The authors have presented the different species or groups of species forming their study system (Lines 85-96). However, there is no clear evidence for the reader why the authors think that the visitation by some species may influence the visitation by other species remain unclear. It would good to set the scene by first talking about "niche partitioning" and "ecological plasticity" that allow ecologically similar species to coexist.

These articles may help:

Salinas-Ramos, V.B., Ancillotto, L., Bosso, L., Sánchez-Cordero, V. and Russo, D. (2020), Interspecific competition in bats: state of knowledge and research challenges. Mam Rev, 50: 68-81. doi:10.1111/mam.12180

Ruczyńki L, Zahorowicz P, Borowik T, Hałat Z (2017) Activity patterns of two syntopic and closely related aerial-hawking bat species during the breeding season in Bialowieza Primaeval Forest. Mammal Research 62: 65– 73.

Schimpp SA, Li H, Kalcounis-Rueppell MC (2018) Determining species specific nightly bat activity in sites with varying urban intensity. Urban Ecosystems 21: 541– 550.

R. – We believe that the evidence for which we consider that the visitation of some species will influence the visitation patterns of another is stated in lines 128-134 of the Introduction section: "*Specifically, we expected that (i) different frugivores species would tend to visit different fruiting individual plants lessening thus interspecific competition or predation risk, (ii) when using the same fruiting plant, prey frugivore species (e.g. small mammals) would tend to temporally avoid plants being foraged by their predators (e.g. carnivores) to reduce predation risk, and (iii) large-bodied frugivores (e.g. ungulates) would facilitate the plant exploitation to smaller frugivores by making the fruit more easily accessible (e.g. by giving up ripe fruits in the plants' immediacy)*".

Also, we have added a paragraph to introduce this issue stating that the visitation patterns of some species may influence other species visitation patterns due to resource-use and niche differences. Many thanks for the references! Which we have included in the text.

L117-125: "*In plant communities, their functional traits vary within environmental gradients and among species occupying similar conditions, raising a challenge for the synthesis of functional and community ecology [36]. The coexistence of consumer species is fostered by resource-use and niche differences, leading to greater resource use in communities with higher number of species [37]. The ecological niche of species is multi-dimensional, including three axes of particular importance that explicitly impact diets and spatial-temporal patterns of abundance: trophic interactions, habitat use and their temporal variability [38]. As a result of interspecific facilitative and competitive interactions, resource partitioning, and niche differentiation, our general hypothesis is…*".

C10. - Line 101: Please clarify in which context the differential visitation of different fruiting plants by different frugivores will contribute to lessening predation

risk. To my knowledge, no species was presented in the system as a predator of another in the same system.

R.- Many thanks for the suggestion. In the introduction section we had added a sentence that introduces this issue:

L103-108: "*This diverse assemblage of frugivores that consume C. humilis and P. bourgaeana's fruits can be grouped into five functional frugivore groups: carnivores, wild ungulates, domestic ungulates, pulp feeders (i.e. lagomorphs and rodents) and birds which may act as seed disperses or seed predators [18, 29-33]; thus, providing a wide spectrum of antagonistic and mutualistic interactions among multiple species across trophic levels [32, 34-35].*".

Following the Editor's suggestions, we have moved the species description to the Methods section and here we show the evidence of predator-prey interactions in our study system (e.g. L174-178: "*In Doñana National Park, C. humilis and P. bourgaeana's seed dispersal is mainly accomplished by medium-size carnivores such as Eurasian badgers (Meles meles) and red foxes (Vulpes vulpes) [28-29]. These two carnivores intensively prey on lagomorphs (Oryctolagus cuniculus) and up to five rodent species (mostly Apodemus sylvaticus and Mus spretus) [32] that feed on C. humilis and P. bourgaeana's pulp and seeds [29-30, 33]*").

C11. - Lines 101-103: As mentioned in my previous comment of line 101, it is only here that it appears that some species are the prey of others in this study system. Please make it clear in the description of the study system (lines 85-96). It would also be good to mention some evidence from the literature showing which species in the system are known to compete for food.

R.- Thanks for the suggestion. As evidence of food competition in our system we included (line 107):

- Valverde, J. A. (1967). Estructura de una comunidad de vertebrados terrestres. Estación Biológica de Doñana. *Monografia*, *1*.

- Fedriani JM, Palomares F, Delibes M (1999) Niche relations among three sympatric Mediterranean carnivores. Oecologia, 121, 138-148.

C12. - Lines 103-105: It is difficult to understand in which ecological context the authors are testing this hypothesis. The authors may provide some evidence or state a theory that will

help understand the relationship between the larger and smaller frugivores.

R. – In order to introduce the reader to the wide spectrum of interactions that we predict, and following the editor's suggestions, we have re-written the description of the study system in the Methods section (lines174-196). Also, we have provided evidence from the literature of the facilitative relationships that we predict (line 194):

- Hernandez, A. (2008). Cherry removal by seed-dispersing mammals: mutualism through commensal association with frugivorous birds. *Polish Journal of Ecology*, *56*(1), 127.
- Fedriani JM, Delibes M (2013) Pulp feeders alter plant interactions with subsequent animal associates. Journal of Ecology, 101, 1581-1588.
- Severo-Neto F, Yule TS, & Costa-Pereira R (2011) Parakeets as facilitators of fish frugivory in the Southern Pantanal. Atualidades Ornitológicas, 169, 15.

Material and methods

C13. - Line 109: Can the author please present a map of the study area so that the reader can quickly have an idea on the scale of the study?

R. – In order to help the reader to have a better acquaintance of the scale of the study, we have added a new Figure (Fig 1) in the revised version; cited in line 138 and line 150; see lines 147-148 for the legend of this Figure) that includes a map of the study area within Europe and the Iberian Peninsula.

C14. - Line 150: In my opinion, this prediction should be in the introduction, and may certainly help understand hypothesis 3, as in the previous comment. Giving evidence from the literature will be important.

R. – Many thanks for the suggestion. Although, in accordance with the Editor's comments we have preferred to leave this prediction as it is in the Methods section. Evidence from the literature about this relation can be found in line 194 (see comment C12, Editor 1)

C15. - Lines 160-163: I would like to know if there any reason why data were not collected for C. humilis at Martinazo in 2019, and why data where not collected for P. bourgaeana in 2018?

R. – Data was not collected for *P. bourgaeana* in 2018 season and *C. humilis* at Martinazo site in 2019 season due to logistic limitations. Please, keep in mind the intense field work effort needed to conduct our investigation.

C16. - Line 163: a) Was there any reference size for each species to be selected in the study sample?
b) Until the end of the document, I have not seen what was the importance of measuring the plant size. Please can you clarify?
R. – We selected fruiting individuals of both plant species regardless of their sizes. Plant size was measured but not used in this study. Thus, we have deleted this information from the revised manuscript (see line 217).

C17. - Line 205: A part of the aim of this research seems to be an example of an analytical approach for the interactions between animal communities and plants. However, the reason for the choice for the main statistical approach (Null model) has not been made clear to the reader. It would be good to know if there exists another method and why the choice for this particular method.
R. – Our intention in this research was to create a new analytical approach to study interactions at a community level. We developed our new methodology (camera trap data + null models) based on re-sampling techniques' literature. The main reason for developing our own null models is because it has enabled us to evaluate the specific hypothesis and predictions that we had projected for our study system. This research could have hardly been able to carry out using other approaches, as they encounter a number of requirements and assumptions that may not fit our specific study system. We have included this reasoning in the Discussion (See Reviewer's 1, comment C21-2).

C18. - Lines 206-2012: Please consider revising. The relationship between the sp1 and the sp2 is not very clear.
R. – We have included the words "by sp2" that might have been missing for a better understanding (line 270 and 272).

C19. - Line 250: For reproducibility of the method, I encourage the authors to indicate the packages used for the analyses, as well as the principal functions.
R. – Indeed. We have added a sentence (lines 316-318) indicating the packages used

for the analysis as well as a supplementary File (S2 file) including the main functions used to tailor the 3 null models.

L316-318: "*The implemented packages used to tailor the null models were plyr [66], dplyr [67] and lubridate [68]. See S2 File for the main functions used in the analysis"*.

Results

C20. - Line 254: When talking about "the most abundant visitors", are you referring to the species with the highest number of visits? If so, please consider revising.

R. – Agree. We have changed this sentence in lines 322-323 referred to *C. humilis:* ("*For the complete set of visits, we found that, in decreasing order, the functional groups with higher number of visits were […]*"). And, also in lines 489-490 referred to *P. bourgaeana: ("For the complete set of visits, we found that, in decreasing order, the functional frugivore groups with higher number of visits were […]"***.**

Discussion

C21. - 1) The results showed the influence of some animal species on the visitation rates of some individual plants by other animal species. However, the authors did not discuss the fact that the spatial locations of individual plants coupled with the spatial distribution of the different animal functional groups may influence such interaction; Although all animal species visited all plant individuals. I suggest discussing this aspect and including it as a limit of the study.

I suggest reading these articles:

García, D., Zamora, R. and Amico, G.C. (2011), The spatial scale of plant-animal interactions: effects of resource availability and habitat structure. Ecological Monographs, 81: 103-121. https://doi.org/10.1890/10-0470.1

Morales, J.M. and Vázquez, D.P. (2008), The effect of space in plant-animal mutualistic networks: insights from a simulation study. Oikos, 117: 1362-1370. https://doi.org/10.1111/j.0030-1299.2008.16737.x

R. – 1) Thank you. We now mention the following (lines 587-594):

"*Other environmental conditions derived from the micro (e.g. plant's location, herbaceous cover, distance to shrub and light availability) and meso habitat (e.g. elevation and type of forest cover) related to each individual plant as well as each animal's niche differences, that allow the coexistence of consumer species, could also exert an effect on the spatial pattern of frugivore visitation [62, 71]. In our system, most of the species (carnivores and ungulates) show high mobility, being able to travel several kilometers within the same day. Therefore, and given the relatively small spatial scale of our study sites, it can be assumed that different species of frugivores had access to all fruiting plants. This scenario however differs for rodents and lagomorphs since they are less mobile; thus, a few cases of spatial avoidance by small mammals identified (null model 1) should be considered with caution* ".

Nevertheless, all species have a wide distribution in our study area and thus we now state (lines 251-253): "*We considered, for each study site, the same pool of vertebrate species due to their high mobility and therefore we did not predict changes in frugivore community structure within each site.*"). However, most of these species (carnivores and ungulates) show high mobility, being able to travel several kilometers in the same day. Therefore, it can be assumed that different species of frugivores had access to the same fruiting plants. Although, we do recognize that this scenario differs for rodents and lagomorphs since they are less mobile".

(Thanks for the references! which have been cited; see line 616.

C21. - 2) Secondly, the authors presented their research using a new approach (combining camera traps and null models). However, they did not discuss their approach with other approaches in the literature and present the advantages of their approach to those in the literature.

R. – 2) See Reviewers 1, comment C17. We included a paragraph in the Conclusion section discussing the advantages that our research can provide to other ongoing studies or methodologies.

Lines 696-711: "*This new approach may provide advantages and complementarities to ongoing research and methodologies for multispecies interactions such as multilayer networks or spatially explicit agent-based simulation modeling [19-20, 89]. For instances, by relaying on data from camera-trapping, our approach can be applied to uncover interspecific interactions among understudied nocturnal or otherwise elusive animals such as many mammals and other secretive vertebrates. Our analytical approach enabled us to evaluate the specific hypothesis and predictions that we had projected for our particular study system and would hardly have been able to achieve*

using other similar approaches (see 26-28) that impose a number of requirements and assumptions that did not always fit our specific system.

This study may also foster a greater understanding of the implications of multispecies plant-animal interactions on ecosystem function and restoration by means of direct and indirect interaction pathways that influence seed dispersal and recruitment in disturbed areas [90]. It can also enrich a great potential of interaction network researches predicting indirect novel interactions or changes in functional network structures under global changing conditions (e.g. land use changes, human disturbance and climate change) [35]."

C22. - Line 542: Change "such" into "such as"

R. – Done. Thanks! See line 568: ("*Large ungulates, such as cows […]*".

Responses to Reviewer #2

C1. - This study presents a framework to evaluate interspecific interactions (attraction, avoidance or indifference) among homeoterms vertebrates across a meta-region harboring population of a palm (Chamaerops humilis) and a flesh-fruited tree species (Pyrus bourgaeana). This assay was based on camera-trapping during two seasons of plant fruiting contrasting the empirical results with three approaches of null models. I have only a few, but rather fundamental, concerns about this study, as below. Moreover, some parts of the paper are hard to understand or unnecessarily long, for this, I suppose that the paper audience will be better attracted to a leaner version. I hope that my suggestions can contribute to the improvement of the manuscript.
R. – We are very thankful to this reviewer for raising his/her concerns. We have taken those inputs into consideration along the revision of the manuscript and we hope that the revised version will clarify the understanding of this study.

Major comments:

C2. - 1) Once your approach is spatially explicit at individual-plant-scale, some environmental characteristics can

affect your results? For example, micro- and meso-habitat features at each plant buffer may bias the results in terms of species interactions. If these factors were not considered or controlled in your analysis, I recommend acknowledging this issue in a paragraph in the discussion section. If you have some environmental or spatial co-variables, I suggest to include that in the regression models to understand the role of environmental features in the influence on vertebrates co-occurrence patterns.

R. – Many thanks for the suggestion. Indeed, there may be some environmental conditions affecting our results (e.g. fruit size, crop size, plant cover). However, our intention in null model 1 analysis was precisely to evaluate whether or not different species of frugivores used the same fruiting plants (independently of the underlying factors). We found that different frugivores species generally used the same individual fruiting trees. Therefore, we then applied null models 2 and 3 to assess potential temporal relationships among frugivore species in the use of such individual fruiting plants. Results of null models 2 and 3 are not affected by each's plant's environmental characteristics as we have based the analysis on time differences between individuals visiting the same fruiting individuals. Therefore, in assessing temporal interactions among frugivores (avoidance, attraction) environmental conditions were rather equal for each pair of frugivore species under investigation.

Furthermore, when we added in our regression models "crop size" as an environmental co-variable, this did not alter the output of the frugivore species interactions. Therefore, for the sake of conciseness, we have omitted these new analyses from the manuscript. Nevertheless, we now mention in the Methods section that patterns derived from model 1 are consistent when crop size is added a covariate.

Methods – Lines 253-258: "*Environmental conditions, related to each plant's micro or meso habitat (e.g. crop size), may affect the visitation patterns of different frugivore species [62] Thus, we assessed the relation between fruit availability (crop size) and total number of visits and interactions by each functional group using Pearson's correlation coefficient using the function corrplot implemented in the R package corrplot [63]* ".

Lines 277-279: "*In a second set of analyses, we added "crop size" as a co-variable in the models. Since the new results did not vary regarding the models without the covariate, for major simplicity, we omitted these results*."

Also, we have added information about the potential effect of environmental variables in the Discussion section:

Lines 612616: “*Other environmental conditions derived from the micro (e.g. plant’s location, herbaceous cover, distance to shrub and light availability) and meso habitat (e.g. elevation and type of forest cover) related to each individual plant as well as each animal’s niche differences, that allow the coexistence of consumer species, could also exert an effect on the spatial pattern of frugivore visitation [62, 71].*”.

C3. - 2) Further, you assumed that all sites have the same pool of vertebrate species? According to your introduction and details in methods, both fruits used by the widest spectrum of consumers, thereby underpinning the trophic structure according to consumer ecological traits (e.g., your functional groups). You can at least discuss this issue because the difference in the local pool of species (real or potential in the future) can cause a rearrangement or bias in the dynamics that you are evaluating.

R. – Thank you. We now discus the effect of local pool of species in the Discussion section, lines 683-686:

“*This outcome may be strongly dependent on initial population densities that would most likely alter the dynamics of the system, thus, the net effect of dispersal success, among other factors, will probably be a result of the abundance of different functional guild of frugivores (legitimate seed dispersers, pulp feeders and seed predators) [87-89].*”.

C4. - 3) If you had data of camera-trapping during two successive resources seasons, what is the reason to only “infer” about the seed dispersal/frugivory? I suggest using the values on fruit removal as a function of species co-occurrence in each plant individual into a regression model. This can reveal an interesting contrast between trees that were patrolled more frequently by predators. I think that this quantitative approach can provide results to your implicit concern (i.e., how much the vertebrate (in)direct interactions influence the frugivory, and potentially the quantitative impacts on seed dispersal and plant fitness).

R. – Many thanks for the suggestion. This is, indeed an interesting approach.

Unfortunately, we do not have fruit removal data as camera-traps cannot record at such level of detail.

C5. - 4) I recognized that you evaluated the visitation according to crop size via correlation, but I suppose that the crop size should be addressed as a co-variable in your regression models to predict the (co)visits. I'm afraid this invalidates the mechanics or the interpretation of much of this very nuanced analysis because the vertebrates' attraction and indifference can be strongly linked to crop-size (as your correlation reveals).

R. –Thank you. As suggested, we added in our regression models "crop size" as an environmental co-variable, this did not alter the output of the species co-occurrence patterns. Therefore, and for the sake of simplicity, we have omitted this information from the manuscript. Nevertheless, we have added information about the potential role of environmental variables in the methodology and discussion of the revised version (see previous response C2-1).

C6. - 4) Once both sites have suffered human-induced alterations, do you have a recommendation derived from your findings for the conservation viewpoint? The novelty of your study is showing an effect of the co-occurrence between frugivores attracted in the space by flesh-fruited plants — an issue little addressed in the literature — but you are assuming a mesocosm equal for all site and plant-individual, for example: (i) the local pool of species; (iii) some environmental features; (iv) past human-induced effects (e.g. disturbance); and (iv) individuals crop-size in each temporal replica.

R. – We consider this is a very interesting question and have included the following in the Conclusion section addressing this issue (lines 706-711: "*This study may also foster a greater understanding of the implications of multispecies plant-animal interactions on ecosystem function and restoration by means of direct and indirect interaction pathways that influence seed dispersal and recruitment in disturbed areas [90]. It can also enrich a great potential of interaction network researches predicting indirect novel interactions or changes in functional network structures under global changing conditions (e.g. land use changes, human disturbance and climate change) [35]*"). But we did not want to speculate too much in the discussion because the intention of this paper is mainly to describe the new methodology and illustrate its applicability with a specific example.

C7. - 5) I identified a disequilibrium between the sections of the manuscript. The manuscript is long but the discussion section is condensed to a few general paragraphs (6 considering the modest conclusion). Whereas the result section needs a rethink about the size and fashion. I regret that some specific comments about them are lacking, but I recommend rethink the results section because it is too long. Some specific results could be synthesized in the result section but better developed their ecological implications in the discussion.

R. –Thank you. This is a good point. We have taken into account this comment throughout the revision of the paper. We have shortened the Results section (See the Editor's comment C9) and expanded our Discussion. For example:

Results: We have merged "Overall patterns" and "null model 1" sections. Also, we have synthesized this section keeping the most important paired interactions (see new Figures 2 and 5) and omitted all 30-minute results, as they were consistent with 5-minute ones (moving the complete outcome to supplementary materials, see S3 File and S3 Table).

Discussion: we now mention, among several other new paragraphs, that:

"*Other environmental conditions derived from the micro (e.g. plant's location, herbaceous cover, distance to shrub and light availability) and meso habitat (e.g. elevation and type of forest cover) related to each individual plant as well as each animal's niche differences, that allow the coexistence of consumer species, could also exert an effect on the spatial pattern of frugivore visitation [62, 71]. In our system, most of the species (carnivores and ungulates) show high mobility, being able to travel several kilometers within the same day. Therefore, and given the relatively small spatial scale of our study sites, it can be assumed that different species of frugivores had access to all fruiting plants. This scenario however differs for rodents and lagomorphs since they are less mobile; thus, a few cases of spatial avoidance by small mammals identified (null model 1) should be considered with caution"* (lines 612-621).

"*Facilitative interactions in the acquisition of food are highly prevalent among many terrestrial vertebrates and have critical importance in structuring and function of many communities [53-55, 76-77]. In contrast with Carreira et al.'s [27] findings, we detected that medium-sized frugivores tended to be temporally attracted by larger ungulates. (…) Our study shows how this functionally diverse assemblage of frugivores leads to interspecific facilitation in foraging, a widespread pattern in other temperate and tropical habitats [55, 76-77]. Positive interactions may be an important mechanism linking high*

diversity to high productivity under stressful environmental conditions [54, 78]" (lines 635-655).

"*As predicted, seed predators and pulp feeders can alter the interaction frequency between fleshy-fruited plants and their mutualistic and antagonistic animal associates [18]. In the first case, ungulate seed predators increased the interaction frequency with legitimate seed dispersers, for both C. humilis and P. bourgaeana yielding thus an indirect positive effect on seed survival (Fig. 7A). Secondly, pulp feeders, that often lessen long-distance dispersal [52], decreased C. humilis' foraging by ungulate seed predators, enhancing thus seed survival and fruit removal by mutualistic animal associates (Fig. 7B)*" (lines 664-670).

"*Therefore, both, mutualistic and antagonistic partners that shape the interaction outcomes among individual plants lead to contrasting seed dispersal success [18, 70], appearing to shape a complex web of direct and indirect effects which's net effect is most likely dependent on the community context [52]. This outcome may be strongly dependent on initial population densities that would most likely alter the dynamics of the system, thus, the net effect of dispersal success, among other factors, will probably be a result of the abundance of different functional guild of frugivores (legitimate seed dispersers, pulp feeders and seed predators) [87-89]*." (lines 680-686).

C8. - 5) Figures: Although I recognize that there were several paired-interaction to show, I suggest that the figures of the manuscript deserve a reformulation to attract the paper audience. I encourage moving the current version of figures (e.g. Fig 1, Fig 2, Fig 5, and Fig 6) to the supporting information files. In doing so, you can select only the most important interactions to show in a new simplified figure avoiding different scales in the y-axis and providing the same color to each species.

R. – Many thanks for this suggestion. We have moved the figures that show all the frugivore paired interactions derived from application of null model 1 (former Figs. 1, 2, 5 and 6) to the supporting information (lines 553-559, S3 File and S3 Table). In order to help the reader to have a better acquaintance of these results, we have merged the most important interaction examples into Figures 2 and 5 (figure description lines 345 and 512, respectively).

Minor comments

C9. - 1) L103-105: "and (iii) large-bodied frugivores (e.g. ungulates) would facilitate the plant exploitation to smaller frugivores by making the fruit more easily accessible (e.g. by giving up ripe fruits in the plants' immediacy)."
I try to understand this presumed facilitation relationship. It seems counter-intuitive because the tendency of large-bodied frugivores ingests the largest portion of fruits. Thus, the interaction expected for the small-bodied frugivores viewpoint is the competition. Do you need to rewrite this premise with more details (as I see in the S1 File).
R. – You're right. We now explain in more detail this facilitative relationship in lines 187-194:

"*Large mammals (e.g. deer, cows) seem to be better adapted in their foraging, either by breaking many of the defensive needle-like spines in fruiting C. humilis or by plucking P. bourgaeana's fruits still attached to the branches (Authors personal observations, see S1 File A). Hence, ungulates remove many fruits of both plant species and often leave some of them whole or partially consumed around mother plants (Authors unpublished data). These left fruits are more easily accessible to medium-sized frugivores which hardly cope with the palm spines and cannot reach the fruits attached to P. bourgaeana branches. Therefore, we predict that the foraging of smaller frugivores could be facilitated by larger ones [e.g., 18, 53-55].*".

C10. - 2) L163-165: "Data on plant size, as well as crop size was recorded for all selected individuals during both study seasons, using sector estimate techniques and if possible total count of fruits."
I strongly suggest referencing methodological sentences such as this example. Several seminal studies adopted this approach (and other approaches, such as lines 193-195). Further, this sentence apparently is repeated in lines 195-197.
R. – Thank you. We have referenced the methods used for crop size estimates (line 218) and also removed the repetition of this information from the manuscript.

C11. - 3) L212-214: "We examined potential significant differences between the average number of visits to PV and PNV in each pair of frugivore species, by fitting Generalized Linear Models with Poisson distribution and log link function."
You need to depict in more detail how these regression models

were performed. Further, I do not understand the reason to use Poisson distribution to continuous data (average number of visits). Is the “average” a count data (sum) to justify the use of Poisson distribution?

R. – Indeed, we have mistaken “average” for “count”. Therefore, for the Generalized Linear Model analysis, we have used as the response variable, the “count” number of visits by one particular species to each plant individual. As a predictor variable, we have used ‘visitation by the other frugivore species with two levels (PV and PNV). We have corrected this information on the manuscript in lines 273-277: “*we examined potential significant differences between the number of visits by one particular species to individual plants visited and not visited by the paired species by fitting Generalized Linear Models with Poisson distribution and log link function [64].”*

C12. - 4) L249-250: “Our analytical approach was performed using free software R 3.5.0 [48].”
Are the functions to perform the null models self-created by you or the analysis was based on a specific R package. Provide more details (but be cohesive) about the data analysis, to this, open space in the manuscript by synthesizing your results.

R. – The functions used to perform the null models are self-created and have been provided to readers in a supplementary appendix (S2 File). As well as for the above reviewer’s request, and for better reproducibility of the method, we have added information about the packages used to create our models. Lines 316-318:

“*The implemented packages used to tailor the null models were plyr [66], dplyr [67] and lubridate [68]. See S2 File for the main functions used in the analysis”.*
